# Multimodal *in vivo* Imaging of the Integrated Postnatal Development of Brain and Skull and Its Co-modulation With Neurodevelopment in a Down Syndrome Mouse Model

**DOI:** 10.3389/fmed.2022.815739

**Published:** 2022-02-11

**Authors:** Sergi Llambrich, Rubèn González, Julia Albaigès, Jens Wouters, Fopke Marain, Uwe Himmelreich, James Sharpe, Mara Dierssen, Willy Gsell, Neus Martínez-Abadías, Greetje Vande Velde

**Affiliations:** ^1^Biomedical MRI, Department of Imaging and Pathology, KU Leuven, Flanders, Belgium; ^2^Grup de Recerca en Antropologia Biológica (GREAB), Department of Biologia Evolutiva, Ecologia i Ciències Ambientals (BEECA), Universitat de Barcelona, Barcelona, Spain; ^3^Center for Genomic Regulation, The Barcelona Institute of Science and Technology, Barcelona, Spain; ^4^Universitat Pompeu Fabra, Barcelona, Spain; ^5^Centro de Investigación Biomédica en Red Enfermedades Raras (CIBERER), Barcelona, Spain; ^6^Institució Catalana de Recerca i Estudis Avançats, Barcelona, Spain; ^7^European Molecular Biology Laboratory (EMBL) Barcelona, European Molecular Biology Laboratory, Barcelona, Spain

**Keywords:** development, brain and skull integration, down syndrome, GTE-EGCG, μCT imaging, μMRI

## Abstract

The brain and skeletal systems are intimately integrated during development through common molecular pathways. This is evidenced by genetic disorders where brain and skull dysmorphologies are associated. However, the mechanisms underlying neural and skeletal interactions are poorly understood. Using the Ts65Dn mouse model of Down syndrome (DS) as a case example, we performed the first longitudinal assessment of brain, skull and neurobehavioral development to determine alterations in the coordinated morphogenesis of brain and skull. We optimized a multimodal protocol combining *in vivo* micro-computed tomography (μCT) and magnetic resonance imaging (μMRI) with morphometric analyses and neurodevelopmental tests to longitudinally monitor the different systems' development trajectories during the first postnatal weeks. We also explored the impact of a perinatal treatment with green tea extracts enriched in epigallocatechin-3-gallate (GTE-EGCG), which can modulate cognition, brain and craniofacial development in DS. Our analyses quantified alterations associated with DS, with skull dysmorphologies appearing before brain anomalies, reduced integration and delayed acquisition of neurodevelopmental traits. Perinatal GTE-EGCG induced disparate effects and disrupted the magnitude of integration and covariation patterns between brain and skull. Our results exemplify how a longitudinal research approach evaluating the development of multiple systems can reveal the effect of morphological integration modulating the response of pathological phenotypes to treatment, furthering our understanding of complex genetic disorders.

## Introduction

It has long been recognized that neural and skeletal development are intimately integrated throughout development (1–3). Although the specific mechanisms are unknown, the interactions between common signaling pathways such as Hedgehog, Wnt, Notch, TGFb and FGF may underlie the tight link between the brain, the face and the skull (2). In pathological conditions affecting brain and craniofacial structures, the most accepted hypothesis is that craniofacial malformations arise because of altered signals emanating from the brain leading to instructional misinformation to the skull (2, 3). However, it could also be possible that defects in craniofacial morphogenesis affect brain development, or that brain and craniofacial dysmorphologies do not induce one another but are produced simultaneously by inappropriate gene expression occurring within each tissue.

The tendency of different traits or systems to vary in a coordinated manner, known as morphological integration, can be assessed by statistical analysis of covariance patterns between phenotypic traits (4). Patterns of morphological integration can reflect genetic, developmental, or functional interactions between anatomical structures (5). In a complex system such as the head, with high interdependence among the brain, cranium and face (1, 2, 6), integration patterns of the head and skull are largely conserved across mammals (7). This shared pattern of skull covariation is usually preserved even under genetic and developmental alterations (8–10).

Morphological integration can influence how the system responds to changes induced by genetic and environmental factors, facilitating or preventing morphological evolution in certain directions of shape change (11). Integration patterns can also modulate the phenotypic output resulting from disease and genetic alterations as those causing rare disorders associated with craniofacial dysmorphologies (12). However, no studies to date have tested how treatments can alter the proper coordination among the different regions of the growing head (8, 10, 12), modulating the dysmorphologies of genetic disorders that present simultaneous brain and craniofacial alterations, such as holoprosencephaly, micro- and macrocephaly, Apert, Pfeiffer and Crouzon craniosynostosis or Down syndrome (DS), the case example in this study.

In DS, a genetic disorder caused by trisomy of chromosome 21, the overexpression of hundreds of genes alters multiple systems and results in intellectual disability, brain malformations and skeletal and craniofacial dysmorphologies (13). Intellectual disability is present in all cases of DS, with varying degrees of severity and including impaired executive function, short-term and working memory and explicit long-term memory (14). The most common gross brain dysmorphologies in DS include a decrease in brain and cerebellar volumes along with an increase in the lateral ventricles' volume (14–17). Simultaneously, craniofacial shape is also altered in DS, showing a brachycephalic shape (with shorter, wider and rounder skulls) and a flattened face with a shorter mandibular ramus (18–20). In this study, we interrogated whether DS is not only affecting multiple systems independently but also their integration and whether there is a co-modulation between dysmorphology and behavioral neurodevelopment impairment.

Interestingly, previous studies have shown that epigallocatechin-3-gallate (EGCG) pure or in enriched green tea extracts (GTE-EGCG) can modulate cognitive performance as well as brain and skeletal development, partly due to its ability to inhibit the kinase activity of the Dual Specificity Tyrosine-(Y)-Phosphorylation Regulated Kinase 1A (DYRK1A), a kinase involved in neural and skeletal development overexpressed in DS (21–27). Accumulating evidence shows that GTE-EGCG can modulate skull morphogenesis in Ts65Dn mouse models (28). In a previous study, we evaluated the effect of high (100 mg/kg/day) or low doses (30 mg/kg/day) of GTE-EGCG, administered from embryonic day 9 to post-natal day 29, on the facial skeletal development in the Ts65Dn DS mouse model (20). Our results identified both negative and positive effects of GTE-EGCG on the craniofacial structure, depending on the dosage, with low doses normalizing craniofacial phenotypes and high doses inducing disparate effects on facial morphology in both trisomic and euploid mice (20). Here we interrogated whether the disruption of normal development produced by high doses of GTE-EGCG alters morphological integration patterns of the brain and the head and how these related to neurobehavioral development, as the treatment may modulate multiple systems simultaneously.

To evaluate the effects of morphological integration patterns on these tightly developing systems, we put forward a novel, multimodal methodological approach. Where most previous studies have focused on either one system or one time point in a cross-sectional design (24, 25, 28–31), we here developed and demonstrated the feasibility of a multimodal imaging-based approach combined with morphometric analyses and parallel neurodevelopmental tests to longitudinally monitor multiple systems' postnatal developmental trajectories simultaneously in live, individual animals from soon after birth into adulthood.

We hypothesize that treatments such as EGCG, affecting both brain and craniofacial development could have an impact on morphological integration patterns. We also propose that alterations to integration may affect early development in DS mouse models. To test this hypothesis, we followed brain and skull postnatal development in parallel in the Ts65Dn mouse model. This holistic approach can help disentangle the direct and indirect effects of potential treatments that regulate altered development in syndromic disorders.

## Materials and Methods

### Animals, Experimental Setup, Treatment and Ethical Statement

Trisomic Ts65Dn (B6EiC3Sn-a/A-Ts (1716)65Dn) mice and euploid littermates were obtained from our in-house breeding colony, established and maintained by crossing Ts65Dn trisomic females to B6EiC3SnF1/J males (refs. 001924 and 001875, the Jackson Laboratory Bar Harbor, ME, USA). Date of conception (E0) was determined as the day in which a vaginal plug was present. Mice were housed at the animal facility of KU Leuven in standard individually ventilated cages (40 cm long × 25 cm wide × 20 cm high) under a 12 h light/dark schedule in controlled environmental conditions of humidity (50–70%) and temperature (22 ± 2°C) with food and water supplied *ad libitum*. All pups were labeled with a non-toxic tattoo ink (Ketchum Animal Tattoo Ink, Green Paste) for identification over longitudinal experiments.

**Figure 1:**
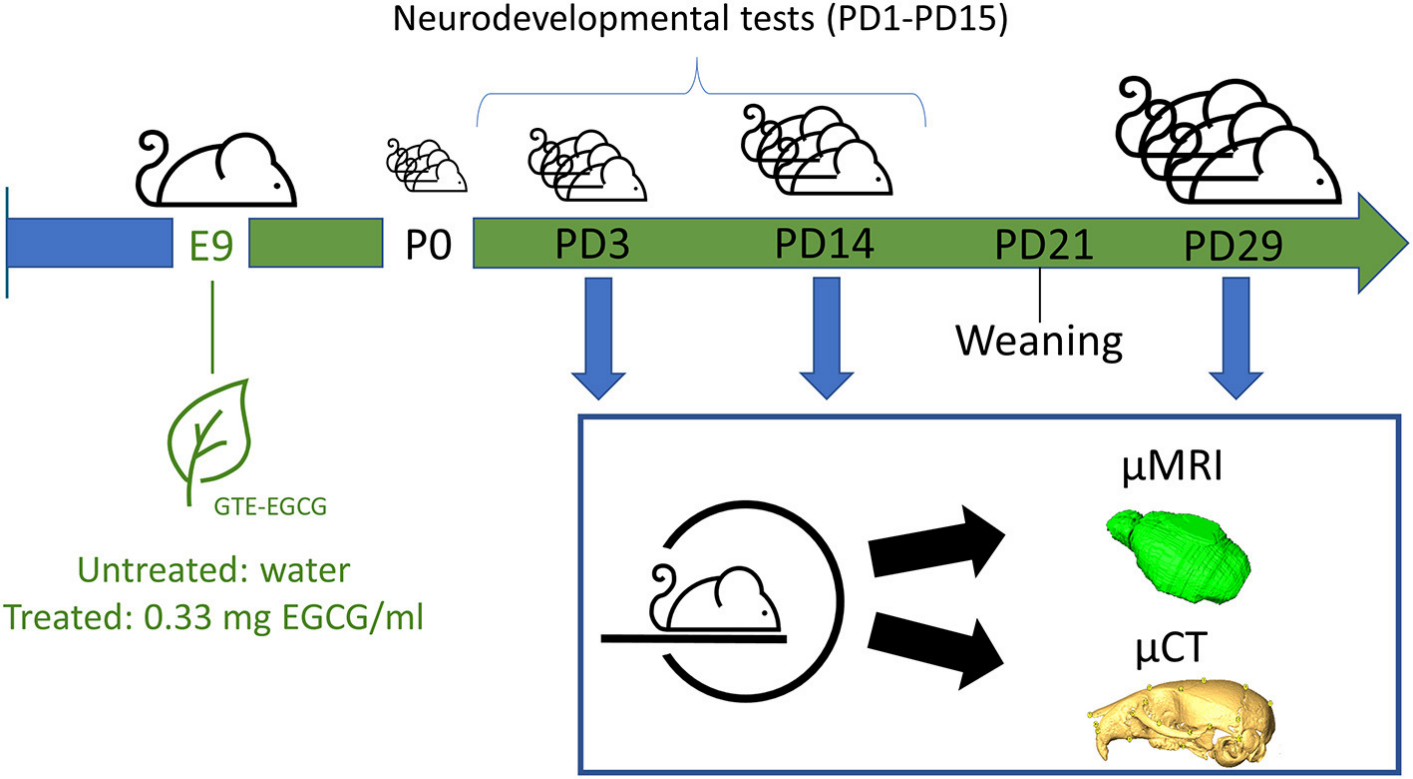
Experimental overview. Ts65Dn mice were scanned *in vivo* from birth to juvenile stages, at three different stages of development [at postnatal day (PD) 3, at PD14 and at PD29] with μCT to longitudinally follow the skeletal development and with μMRI to follow brain development. Pups were tested daily from PD1 until PD15 for the appearance of developmental traits and reflexes related with their neurobehavioral development. Half of the litters were treated with GTE-EGCG at a concentration of 0.33 mg EGCG/mL resulting in a dose of ~100 mg/kg/day.

We bred a total of 7 litters, three of which were administered a treatment with GTE-EGCG [Mega Green Tea Extract, Life Extension, USA; with estimated composition of 53.6% EGCG, 4.5% epicatechin (EC), 9% epicatechin gallate (ECG) and 12.5% epigallocatechin (EGC)] via the drinking water at a concentration of 0.33 mg EGCG/mL. As EGCG crosses the placenta and reaches the embryo (32), GTE-EGCG treatment started prenatally at embryonic day 9 (E9) via the drinking water of the pregnant dams and continued until the end of the experiment. After weaning, from P21 to P29, GTE-EGCG dissolved in water at the same concentrations was made available to the young mice *ad libitum* (Figure 1). The dosage of EGCG received for an adult mouse was ~100 mg/kg/day, considering that, on average, early adult mice weigh 20 g and drink 6 mL of water per day, according to our measurements. In developing embryos and pups before weaning, the received dosage was lower since previous studies indicate that maternal plasma concentrations of catechins were about 10 times higher than in placenta and 50–100 times higher than in the fetal brain (32, 33) and EGCG in milk and plasma of postnatal day 1 (PD1) to PD7 pups was detected at low concentrations (30). Three litters of mice were treated following this protocol and four litters were left untreated for comparison. To minimize potential batch effects, the treatment for all mice was prepared freshly every 3 days from the same batch of GTE-EGCG and water intake was monitored in each cage.

Mice were genotyped at PD14 by PCR from ear snips adapting the protocol in Shaw et al. (34). Trisomic primers, Chr17fwd-5′-GTGGCAAGAGACTCAAATTCAAC-3′ and Chr16rev-5′-TGGCTTATTATTATCAGGGCATTT-3′; and positive control primers, IMR8545-5′-AAAGTCGCTCTGAGTTGTTAT-3′ and IMG8546-5′-GGAGCGGGAGAAATGGATATG-3′ were used. The following PCR cycle conditions were used: step 1: 94°C for 2 min; step 2: 94°C for 30 s; step 3: 55°C for 45 s; step 4: 72°C for 1 min (steps 2–4 repeated for 40 cycles); step 5: 72°C for 7 min, and a 4°C hold. PCR products were separated on a 1% agarose gel. Randomization for genotype was performed by testing all the pups in each litter, which contain both WT and TS mice. Treatment was randomized by treating half of the litters.

Mice were allocated to groups according to their genotype and pharmacological intervention: wild-type (WT) and trisomic Ts65Dn (TS) mice untreated or treated with GTE-EGCG. During experimentation the investigators were blinded to genotype but not to treatment since they administered the treatment. During data analysis, all investigators were blinded to both genotype and treatment. The sample size for each experiment is described in Supplementary Table S1. Differences in sample size are due to technical reasons such as missing scan because the micro-CT or MR scanner was not operating on the scanning day, movement scanning artifacts that impaired image quality, or mouse death during the experiment. Moreover, during the data analyses, few individual mice were excluded when identified as outliers by the ROUT test (35) using GraphPad Prism (v8.02, GraphPad Software, San Diego, California USA) with a Q (maximum desired False Discovery Rate) of 1%. The number of outliers per group is specified in Supplementary Table S1.

All procedures complied with all local, national and European regulations and ARRIVE guidelines and were authorized by the Animal Ethics Committee of KU Leuven (ECD approval number P004/2016).

### *In vivo* Micro-Magnetic Resonance Imaging

Mice were anesthetized by inhalation of 1.5–2% of isoflurane (Piramal Healthycare, Morpeth, Northumberland, United Kingdom) in pure oxygen. *In vivo* anatomical μMR images were acquired through a 3D T2 weighted RARE sequence (TR/TE: 1,000/ 60 ms; RARE factor: 16; FOV: 24 × 15 × 8.3 mm3; matrix 256 × 160 × 88; acquisition time 15 min) on a 9.4 T Bruker Biospec small animal μMR scanner (Bruker Biospin, Ettlingen, Germany; 20 cm horizontal bore) equipped with actively shielded gradients (600 mT m^−1^). A dedicated 3D printed holder was used to accommodate the pups at PD3 and PD14 and to minimize movement artifacts. A quadrature radio-frequency resonator (inner diameter 7.2 cm, Bruker Biospin) was used for transmission of radiofrequency pulses and decoupled to a brain surface coil (quadrature shaped surface coil optimized for mouse brain scanning, Bruker Biospin).

### *In vivo* Micro-Computed Tomography

After μMRI, mice were anesthetized and scanned *in vivo* with the SkyScan 1278 (Bruker Micro-CT, Kontich, Belgium) for 3 min using the optimized parameters specified in Supplementary Table S2.

### Image Data Processing

To segment the whole brain and the ventricles from the μMR images, data were first converted to Analyze file format using ImageJ (1.52 d, National Institute of Health) and then loaded into Amira 2019.3 (Visualization Sciences Group, FEI). Brain and ventricles were manually segmented by drawing a region of interest (ROI) covering those structures on each image slice. Segmentations were used to generate 3D models and to estimate the associated volume.

To segment the craniofacial complex, μCT data was first reconstructed (NRecon software, Bruker Micro-CT, Kontich, Belgium) and then loaded into Amira 2019.3 (Visualization Sciences Group, FEI). 3D models of craniofacial structures were automatically generated by creating an isosurface based on specific threshold for bone.

### Size Analysis

To evaluate if growth trajectories of brain and ventricular structures were significantly different between groups, we analyzed the data by fitting a mixed model as implemented in GraphPad Prism 8.0, with the Geisser-Greenhouse correction. This mixed model uses a compound symmetry covariance matrix and is fit using Restricted Maximum Likelihood (REML). In the presence of randomly missing values, the results can be interpreted like a repeated measures ANOVA. In these analyses, three factors were analyzed, time (reflecting growth across different stages), group (reflecting either genotype or treatment differences at baseline, depending on the groups analyzed) and the interaction between time and group (reflecting differences in the growth patterns). Since time resulted always significant as all groups of mice grew and increased their volume over the different stages analyzed, we focused on differences in the growth patterns as reflected by the interaction effect. Then, since the data were not normally distributed according to Shapiro-Wilk tests, Mann Whitney tests were performed between each pair of experimental groups (WT vs. TS, WT vs. WT Treated, WT vs. TS Treated, TS vs. WT Treated, TS vs. TS Treated, TS Treated vs. WT Treated) at each timepoint to assess if the volumes of the brain and the ventricles were significantly different between groups within each stage using GraphPad Prism (v8.02, GraphPad Software, San Diego, California USA). Only the *P*-values that were classified as discoveries according to the Benjamini–Hochberg correction for multiple comparisons (Q = 5%) are reported.

### Shape Analysis

We compared the morphology of brain and craniofacial structures in WT and TS mice with and without GTE-EGCG treatment using quantitative shape analyses (36). Geometric Morphometrics (GM) is a sophisticated body of statistical tools developed for measuring and comparing shapes with increased precision and efficiency (11, 37). The analysis was based on the 3D coordinates of anatomical homologous landmarks recorded over the structures of interest at each developmental stage, as defined in Supplementary Figure S1 and Supplementary Tables S3–S5. Landmarks were acquired using Amira 2019.3 (Visualization Sciences Group, FEI) and all the GM analyses were performed using MorphoJ v1.06d (38).

To extract shape information from the 3D coordinates of the landmark configurations we performed a Generalized Procrustes Analysis (GPA), which minimizes the influence of size and adopts a single orientation for all specimens. After landmark superimposition, we performed a Principal Component Analysis (PCA), a data exploration technique that performs an orthogonal decomposition of the data and transforms variance covariance matrices into a smaller number of uncorrelated variables called Principal Components (PCs), which successively account for the largest amount of variation in the data (39). Shape variation was represented by creating a morphospace based on the two first PCs, which explain the largest percentages of morphological variation within the sample. If there are no dysmorphologies associated with DS or the treatment, WT and TS mice, treated or not, will overlap in the PCA scatterplot, showing similar phenotypes. If there are differences due to genotype or treatment, groups of mice will be separated from each other, showing a distinct phenotype. PCA results were plotted with the ggpubr package in R (40, 41).

From the 3D coordinates of the brain landmarks we estimated additional brain measures. The cephalic index was calculated as the ratio of the Euclidean distance between landmarks number 4 and 5 and landmarks number 1 and 3 (1 and 11 at PD3) of the brain. The length of the olfactory bulbs and the length of the cerebellum were determined as the Euclidean distance between landmarks number 1 and 2 and 10 and 3, respectively (see Supplementary Table S3 for landmark definitions).

To statistically quantify overall differences between WT, TS, WT treated and TS treated mice, we estimated the Procrustes distances between the average shapes of pairs of groups at each stage of development. Procrustes distances are used in GM to quantify group shape differences after the process of superimposition and are calculated as the square root of the sum of squared differences in the positions of the landmarks in the two average shapes. Permutation tests based on the Procrustes distances were used to estimate the associated *P*-values and to assess the statistical significance of shape differences between groups.

To further evaluate whether more severe brain malformations were associated with more severe skull malformations, we estimated brain and skull deviance as the Procrustes distance between the brain and skull of each mouse and the average shape of the WT untreated group. Then, we calculated the Pearson's coefficients per group and plotted the results using the ggpubr package in R (40, 41).

Finally, brain and skull integration was evaluated analyzing the covariation patterns of the brain and skull shape during development by performing a two-block partial least square (PLS) analysis (42). This method performs a singular value decomposition of the covariance matrix between the two blocks of shape data (i.e., the brain and the skull data). Uncorrelated pairs of new axes are derived as linear combinations of the original variables, with the first pair accounting for the largest amount of inter-block covariation, the second pair for the next largest amount and so on. The degree of covariation is measured by the RV coefficient, a multivariate analogue of the squared correlation (43). Statistical significance was tested using permutation tests under the null hypothesis of complete independence between the two blocks of variables. The results were plotted and the Pearson's coefficients for correlation between the two blocks were calculated for each group using the ggpubr package in R (40, 41).

### Developmental Tests

Testing for neurobehavioral development was carried out daily from PD1 to PD15 as previously described (44). Pups were kept in their home cage and were taken out of the cage one at a time for testing whereas the mother was separated before testing. Once all pups were evaluated, mothers were put back in the cage. The day of appearance of developmental landmarks was recorded and used as the unit of analysis. Neurobehavioral analysis included a battery of tests evaluating pre-weaning sensorial responses that reflect the maturation of the central nervous system. During the experiment, tactile startle response (blast response; a strong blow causes an immediate startle response, a sudden extension of the head and fore and hindlimbs, which are then withdrawn and a crouching position assumed), vibrissae placing (when the mouse is suspended by the tail and lowered so that the vibrissae contact a solid object, the head is raised and the fore limbs are extended to grasp the object), tactile orientation, opening of the eyes, detachment of the pinna and incisor eruption were evaluated. The first day in which the reflexes or landmarks appeared was recorded.

Since sex differences were not significant, we pooled data from male and female mice for analysis.

Developmental tests were analyzed with a logrank test (Mantel-Haenszel approach), where the day of appearance of the landmark or reflex was introduced as an event using GraphPad Prism.

## Results

We developed a combination of *in vivo* micro computed tomography (μCT) and magnetic resonance imaging (μMRI) to investigate the skeletal and brain development over postnatal developmental through quantitative morphometric analysis in parallel with a battery of developmental tests to evaluate the neurobehavioral development (Figure 1). For our case study, we used the Ts65Dn (TS) Down syndrome mouse model, which is a widely studied DS mouse model (45, 46) that carries a segment with ~120 genes homologous to Hsa21 (starting upstream of *Mrpl39* to the telomeric end of Mmu16), translocated to a small centromeric part of Mmu17 (47–49). These mice are trisomic for about two-thirds of the genes orthologous to Hsa21, but also carry genes originating from the Mmu17 that are not related to DS disease, including about 46 protein-coding genes, 35 nonprotein-coding genes and 35 pseudogenes (50). These genetic alterations do not fully represent Down syndrome's aneuploidy, but recapitulate the main craniofacial, brain and neurodevelopmental alterations associated with DS (20, 21, 51–53). We experimentally evaluated the effect of a green tea extract (GTE-EGCG; Mega Green Tea Extract, Life Extension, USA) on these systems during pre- and postnatal development of mice. The GTE-EGCG solution was prepared freshly using the same batch of the Mega Green Tea Extract in all the experiments, with an estimated composition of 53.6% EGCG, 4.5% epicatechin (EC), 9% epicatechin gallate (ECG) and 12.5% epigallocatechin (EGC).

### Postnatal Brain Anatomy Alterations in Ts65Dn Mice and Modulatory Effects of Perinatal GTE-EGCG Treatment

**Figure 2:**
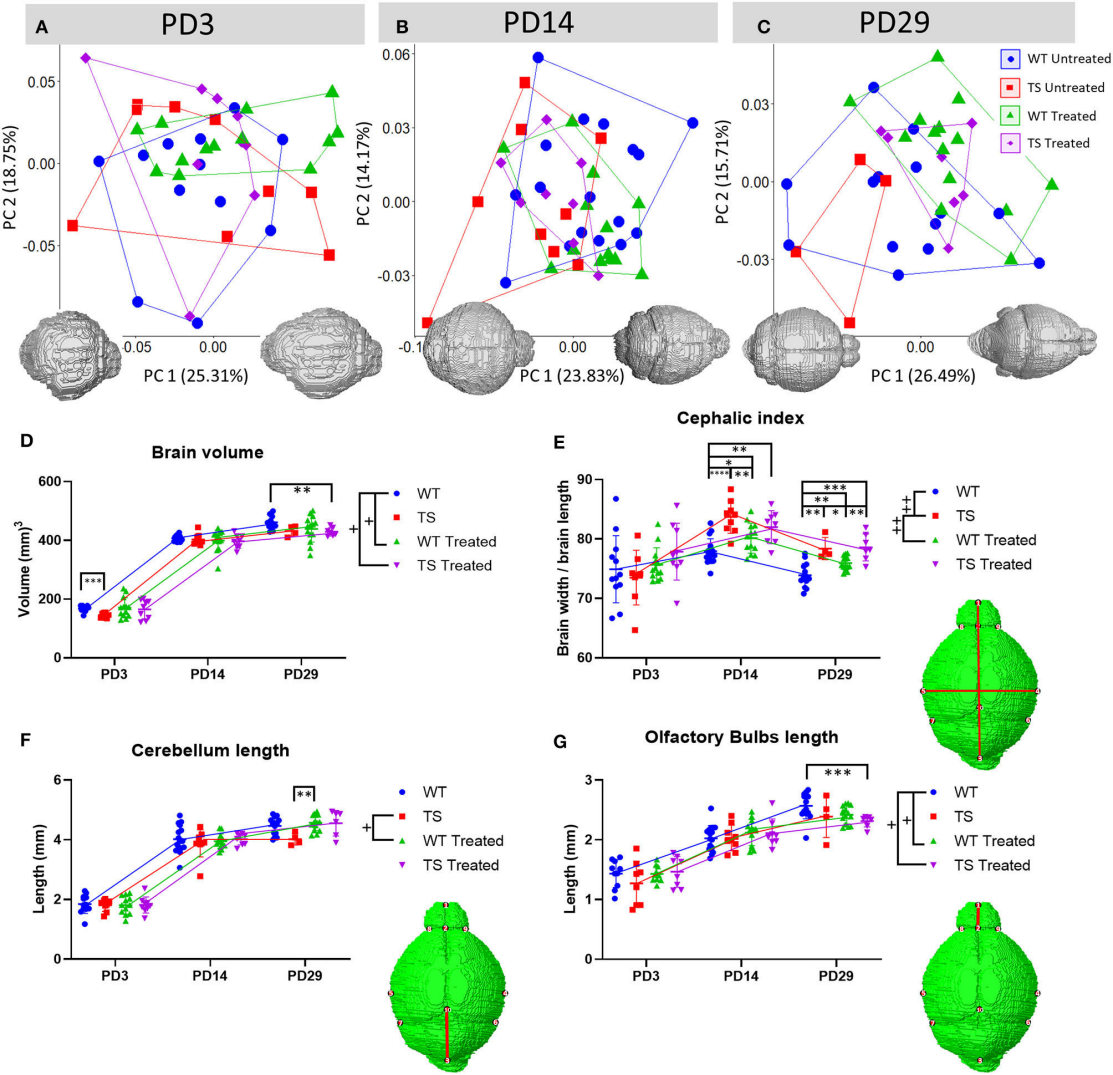
Postnatal brain anatomy alterations in Ts65Dn mice and modulatory effects of perinatal GTE-EGCG treatment. **(A–C)** Brain shape variation assessed by a Principal Component Analysis (PCA) based on landmarks registered on the 3D brain reconstructions segmented from *in vivo* μMRI scans at **(A)** PD3, **(B)** PD14, and **(C)** PD29. Each symbol represents one mouse. All scatter plots are presented along with the morphings associated with the negative and positive values of the PC1 axis. Morphings represent shape changes associated with PC scores of 0.1 and −0.1. **(D)** Brain volume, **(E)** cephalic index (estimated as the ratio between the width and length of the brain), **(F)** length of the cerebellum and **(G)** length of the olfactory bulbs at PD3, 14 and 29. Data are presented as mean ± standard deviation. See Supplementary Figure S1 and Supplementary Table S3 for more details about how lengths and distances for the brain structures were estimated. ^+^*sp* < 0.05; ^++^*p* < 0.01; Mixed-effects analysis across timepoints; ^*^p < 0.05; ^**^p < 0.01; ^***^p < 0.001; ^****^p < 0.0001; Mann Whitney tests by timepoint. Sample size: (PD3) WT = 12 (10 olfactory bulbs), TS = 8, WT Treated = 13 (12 cerebellum length, 11 olfactory bulbs), TS Treated = 9 (nine olfactory bulbs); (PD14) WT = 17, TS = 9, WT Treated = 13, TS Treated = 8; (PD29) WT = 14, TS = 4, WT Treated = 14, TS Treated = 7 (six cerebellum length). The mice analyzed may differ across stages but represent overall ontogenetic trajectories.

In the first series of experiments, we defined the postnatal trajectories of brain development and the effects of a perinatal 100 mg/kg/day GTE-EGCG treatment. At PD3, the shape analysis based on brain MRI indicated that overall brain morphology, defined by a set of 10 brain landmarks, was similar between WT and TS mice, as the two groups overlapped on the morphospace created by the first two Principal Components, which explained 44.06% of total morphological variance (Figure 2A). Permutation tests based on the Procrustes distance between WT and TS mice, confirmed that their brain shape was not significantly different at this early stage (*P*-value = 0.5553) (Supplementary Table S6). The brain shape differences between WT and TS mice developed over time and became significant by PD14 (*P*-value = 0.0095; Figure 2B). In comparison to WT mice, TS mice presented shorter and wider brains at PD14. However, by PD29 interindividual morphological variation increased and shape differences were no longer significant at this last stage (*P*-value = 0.0519) (Figure 2C; Supplementary Table S6).

Regarding GTE-EGCG treatment effects, perinatal 100 mg/kg/day GTE-EGCG supplementation negatively affected the control group at the earliest and latest stages, as even though there was some overlap between WT treated and WT untreated mice in the PCA (Figures 2A,C), the two groups were significantly different at PD3 (*P*-value = 0.026) and PD29 (*P*-value = 0.009). TS treated mice did not show any significant effect of GTE-EGCG treatment at PD3 and PD14, but by PD29, they no longer overlapped with TS untreated mice and presented a significantly different brain shape than TS untreated (*P*-value = 0.011) and WT untreated mice (*P*-value = 0.016) (Figure 2C; Supplementary Table S6).

Regarding brain volume, a mixed-effects analysis revealed no significant differences in the growth trajectories of TS untreated mice as compared to WT untreated mice (*P*-value = 0.0597) (Figure 2D). The Mann Whitney tests indicated that TS mice presented smaller brains at PD3 (*P*-value = 0.0003). However, there was a period of intense growth from PD3 to PD14, in which both WT and TS mice increased their brain size and TS mice caught up in their growth, so that no genotype-dependent differences were detected at PD14 and PD29 (Figure 2D).

GTE-EGCG treatment altered the brain growth trajectory of WT treated mice (*P*-value 0.0393) (Figure 2D). At PD3, perinatal 100 mg/kg/day GTE-EGCG increased the brain size of several WT and TS treated mice, resulting in TS treated mice not being significantly different than WT untreated mice (*P*-value = 0.917). However, since this rescuing effect was not achieved in all treated mice, TS treated mice were not significantly different than TS untreated mice (*P*-value = 0.3088). This effect of the treatment was not detected at later stages. In fact, at PD29, TS treated mice presented smaller brains than WT untreated mice (*P*-value= 0.0023) (Figure 2D).

Besides overall brain shape, we also assessed specific head and brain traits that are typically associated with DS. We estimated the ratio of brain width and length (cephalic index), as a proxy of head brachycephaly, a common dysmorphology in DS associated with a shorter and wider head (18, 54) (Figure 2E). The comparison of cephalic indexes over development reflects how the brain proportions of length and width change, until reaching adult final size and shape (Figure 2E). The mixed-effects analysis indicated that, as compared to WT untreated mice, TS untreated mice presented a significantly different brain growth pattern (*P*-value = 0.0049). The Mann Whitney tests revealed disproportionately higher cephalic indexes in TS untreated mice than WT untreated mice by PD14 (*P*-value < 0.0001), and PD29 (*P*-value = 0.0026) (Figure 2E). GTE-EGCG treatment did not have any effect in TS mice, as they maintained high cephalic indexes, but increased the cephalic index in WT treated mice as compared with WT untreated mice at PD14 (*P*-value = 0.0154) and PD29 (*P*-value = 0.0058) (Figure 2E).

We also assessed alterations in the cerebellum, as this is one of the most affected brain regions in DS (55, 56). The mixed-effects analysis revealed significant differences in the growth trajectories only between WT treated and TS untreated mice (*P*-value = 0.0271), specifically at PD29 (*P*-value = 0.0026, Mann Whitney tests) (Figure 2F).

No genotype differences were found in the olfactory bulbs, but GTE-EGCG modified the growth trajectory in WT treated mice (*P*-value = 0.0403). No significant effects were detected in TS treated mice as compared to TS untreated mice, but TS treated mice showed a significantly different growth pattern (*P*-value = 0.0392) and a reduced length at PD29 (*P*-value = 0.0008) as compared to WT untreated mice (Figure 2G).

### Postnatal Ventricular Alterations in Ts65Dn Mice and Modulatory Effects of Perinatal GTE-EGCG Treatment

**Figure 3:**
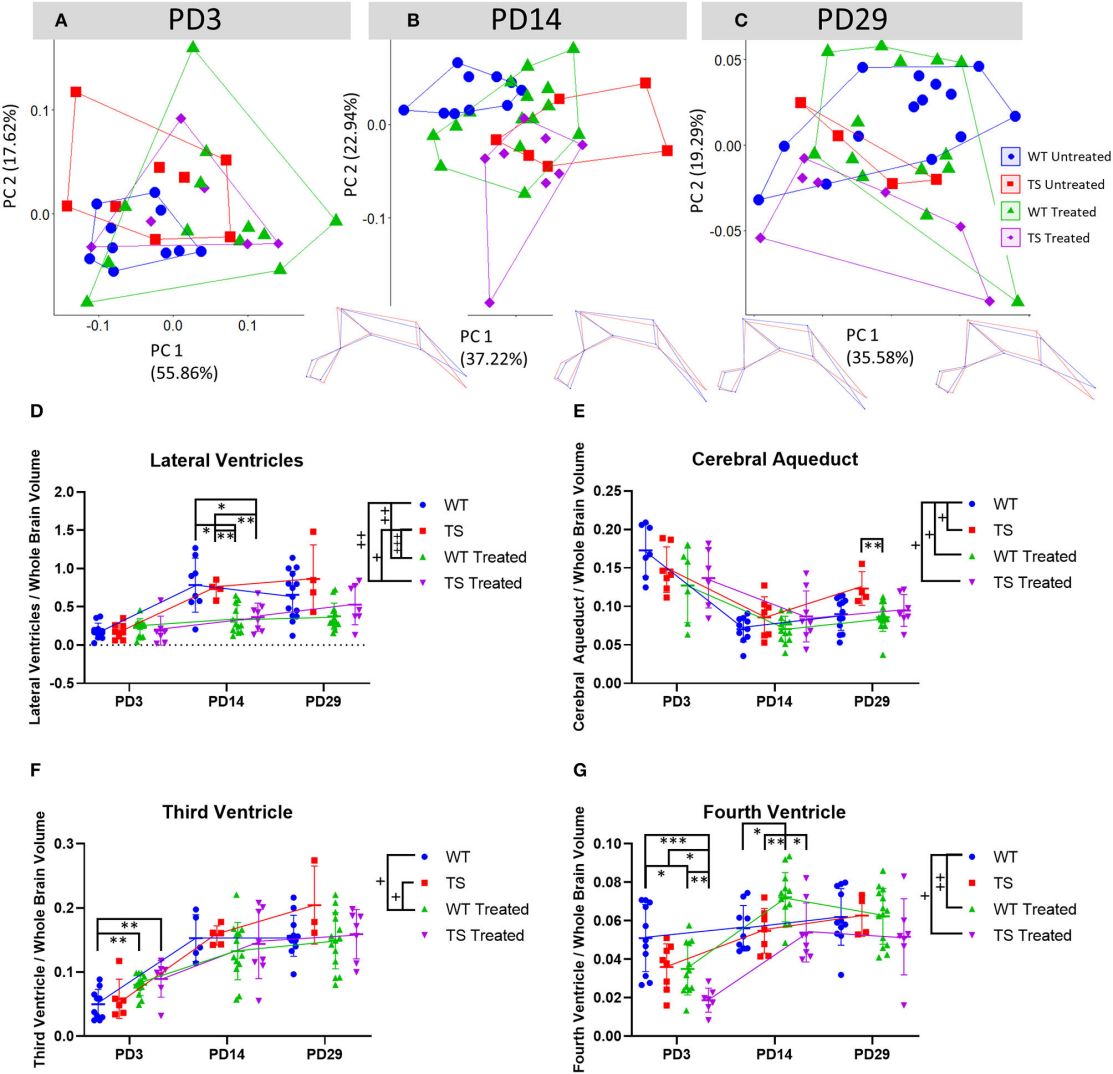
Postnatal ventricular alterations in Ts65Dn mice and modulatory effects of perinatal GTE-EGCG treatment. **(A–C)** Ventricular shape variation assessed by a Principal Component Analysis (PCA) based on the landmarks registered on the sagittal plane of *in vivo* μMRI scans as described in Supplementary Table S4 at **(A)** PD3, **(B)** PD14, and **(C)** PD29. PCA are presented along with the wireframes displaying ventricular morphologies along PC1. Dark blue wireframes represent the morphology associated with the specimens located on the negative or positive extreme of PC1 (shape changes associated with PC scores of 0.1 and −0.1) in comparison to the mean shape of the sample (red wireframe) to better illustrate the differences in shape. Ratios of the volumes of **(D)** the lateral ventricles, **(E)** the cerebral aqueduct, **(F)** the third ventricle and **(G)** the fourth ventricle normalized by the total brain volume at postnatal days (PD) 3, 14, and 29, estimated from *in vivo* μMRI scans. Data are presented as mean ± standard deviation. ^+^*p* < 0.05; ^++^*p* < 0.01; ^+++^*p* < 0.001; Mixed-effects analysis across timepoints; ^*^p < 0.05; ^**^p < 0.01; ^***^p < 0.001; Mann Whitney tests by timepoint. Sample size: (PD3) WT = 11 (10 third ventricle and ventricular shape, seven cerebral aqueduct), TS = 8 (seven cerebral aqueduct, six third ventricle), WT Treated = 12 (11 lateral ventricles, six cerebral aqueduct), TS Treated = 7 (six cerebral aqueduct and ventricular shape); (PD14) WT = 10 (nine fourth ventricle, eight lateral ventricles, five third ventricle), TS = 7 (six ventricular shape, five third ventricle, four lateral ventricles), WT Treated = 13 (15 ventricular shape, 12 lateral ventricles), TS Treated = 8; (PD29) WT = 14 (13 cerebral aqueduct, 11 third ventricle and fourth ventricle), TS = 4 (three third ventricle), WT Treated = 14 (13 lateral ventricles), TS Treated = 7.

Next, since ventricular anomalies are usually associated with DS (15–17, 57, 58), we evaluated the volume and shape of the ventricles, as defined by a set of nine landmarks located on the ventricles. At PD3, the shape of the ventricular system was similar between all groups of mice, as indicated by the overlap between groups at the PCA (Figure 3A). However, WT treated mice showed a disparate range of morphological variation, with most mice falling outside the range of variation of WT untreated mice (Figure 3A). Permutation tests revealed significant differences between untreated and treated WT mice (*P*-value = 0.017), indicating that 100 mg/kg/day perinatal GTE-EGCG treatment significantly altered WT treated mice at this early stage. At PD14, all groups were significantly different from each other (Supplementary Table S7), indicating both genotype and treatment effects (Figure 3B). At PD29, however, the morphological differences were reduced, and all groups overlapped except for TS treated mice (Figure 3C), which showed a significantly different ventricular shape compared to WT untreated mice (*P*-value = 0.026) but were not different from TS untreated mice (*P*-value = 0.387).

Although ventriculomegaly has often been associated with DS (15–17, 57, 58), we did not find larger lateral ventricles over postnatal development in TS untreated mice (Figure 3D). Only at PD29, TS untreated mice tended to present larger lateral ventricles, but this difference did not reach significance (*P*-value = 0.5052). TS untreated mice showed a significantly different growth pattern in the cerebral aqueduct (*P*-value = 0.0203) (Figure 3E), but no significant differences in the volume of the third (Figure 3F) and fourth ventricle (Figure 3G).

Our analysis suggested that GTE-EGCG had diverse effects on the volume of the ventricles depending on the ventricular structure and developmental time. The mixed-effects analysis revealed that GTE-EGCG treatment significantly modulated the growth trajectories of the lateral ventricles of WT treated (*P*-value = 0.0015) and TS treated mice (*P*-value = 0.0260) (Figure 3D). Specifically, the Mann Whitney tests showed that GTE-EGCG decreased the volume of the lateral ventricles in both WT treated (*P*-value = 0.0302) and TS treated (*P*-value = 0.0031) mice at PD14 (Figure 3D). The mixed-effects analysis detected significant differences between WT treated and WT untreated mice in the growth trajectories of the cerebral aqueduct (*P*-value = 0.0223), third (*P*-value = 0.0259) and fourth ventricle (*P*-value = 0.0018) (Figures 3E–G). Perinatal 100 mg/kg/day GTE-EGCG significantly increased the volume of the third ventricle in WT treated mice at PD3 (*P*-value = 0.0071) (Figure 3F), reduced the volume of the fourth ventricle in WT treated (*P*-value = 0.0317) and TS treated (*P*-value = 0.014) mice at PD3, increased the volume of the fourth ventricle in WT treated mice (*P*-value = 0.0111) at PD14 and had no effect at PD29 (Figure 3G).

### Craniofacial Alterations in Ts65Dn Mice Are Not Rescued by Perinatal GTE-EGCG

**Figure 4:**
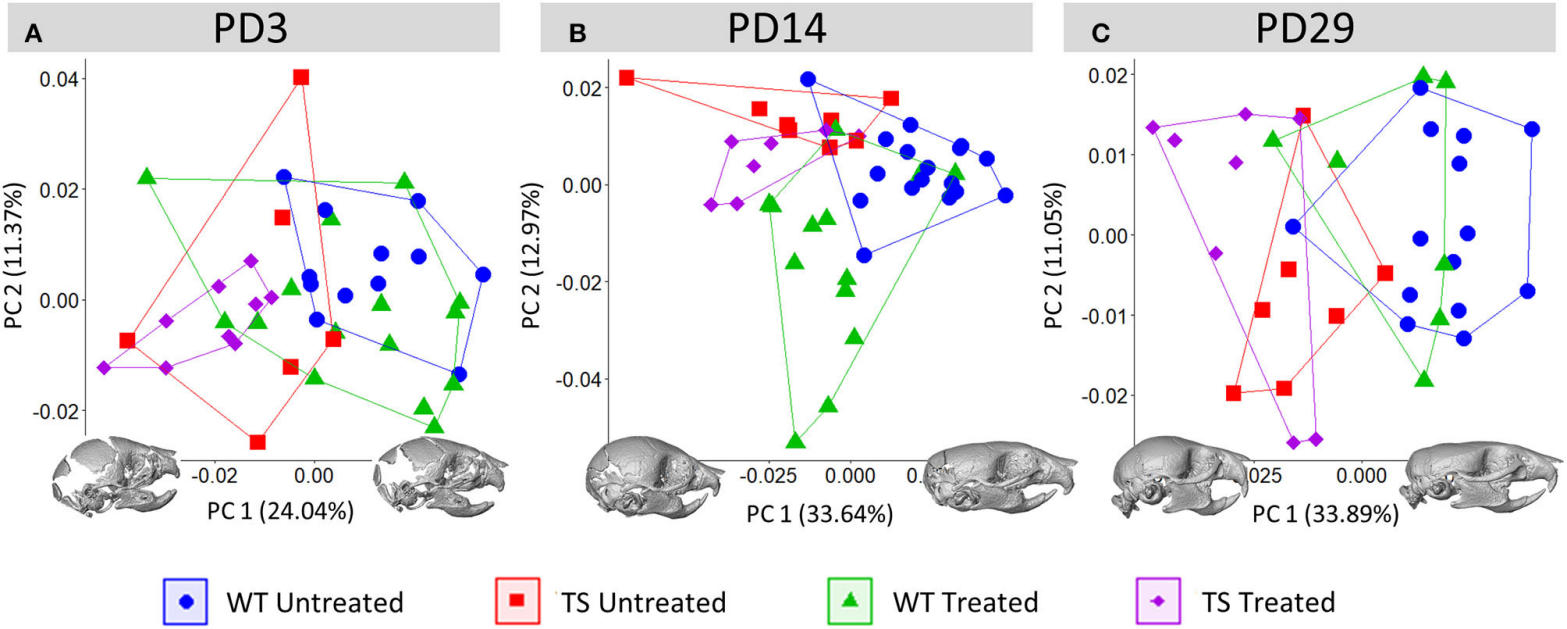
Development of the postnatal skull and facial shape development and effects of GTE-EGCG. Skull shape variation as assessed by a Principal Component Analysis (PCA) based on the 3D coordinates of landmarks placed on the surfaces of 3D renders from *in vivo* μCT scans as described in Supplementary Table S5 at **(A)** PD3, **(B)** PD14 and **(C)** PD29. All scatter plots are presented along with the morphings associated with the negative and positive values of the PC1 axis. Morphings are exaggerated to represent a shape associated with a PC score of 0.08 and −0.08. Sample size: (PD3) WT = 12, TS = 6, WT Treated = 15, TS Treated = 9; (PD14) WT = 17, TS = 8, WT Treated = 13, TS Treated = 7; (PD29) WT = 14, TS = 7, WT Treated = 7, TS Treated = 8.

To evaluate the development of craniofacial morphology over time we used the μCT images acquired in parallel to the *in vivo* μMR images with which we assessed brain development. Our quantitative shape analyses indicated that the morphological differences between the skull of WT and TS untreated mice were already significant at PD3 (*P*-value = 0.0042) and that these shape differences were maintained at PD14 (*P*-value <0.0001) and PD29 (*P*-value = 0.0003) (Figures 4A–C). TS untreated mice showed shorter, wider and more globular skulls, as compared to elongated and flat skulls typically associated with WT untreated mice (Figures 4A–C).

Craniofacial malformations were not rescued by perinatal 100 mg/kg/day GTE-EGCG treatment at any stage, as TS treated mice never overlapped with WT untreated mice (Figures 4A–C). On the contrary, GTE-EGCG supplementation altered skull development as WT and TS treated mice were displaced away from their untreated counterparts in the PCA, with TS treated mice occupying the most extreme position in the morphospace in comparison to WT untreated mice at PD29 and showing the most dysmorphic shapes (Figure 4C). Results from the permutation tests indicated that these differences were not significant in TS treated mice but confirmed the shape changes in WT treated mice at PD14 and PD29 (Supplementary Table S8).

### Correlation Between Brain and Skull Dysmorphology Altered by Genotype and Perinatal GTE-EGCG Treatment

**Figure 5:**
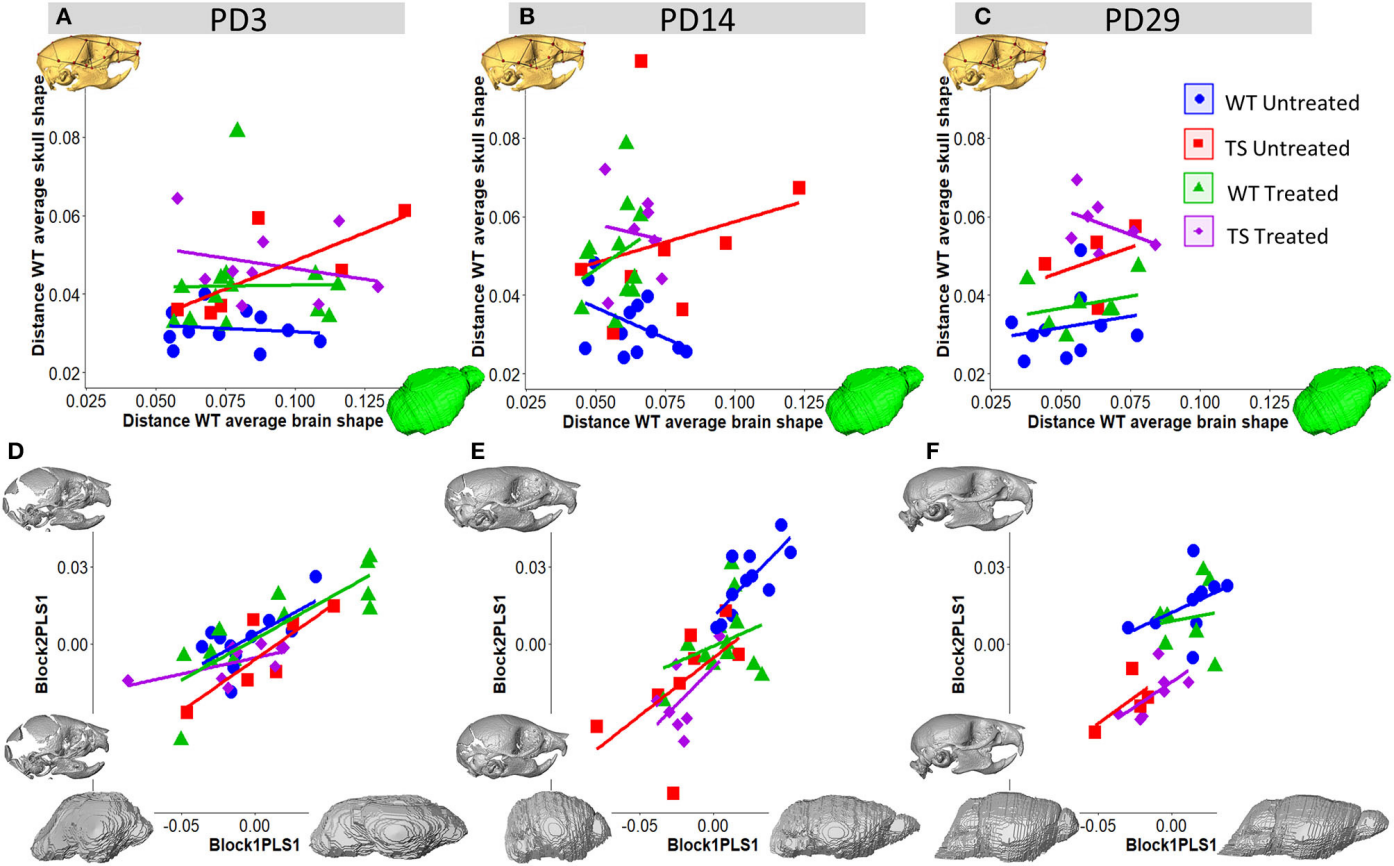
Skull and brain integration over postnatal development in wild-type and Ts65Dn mice, and effects of GTE-EGCG. Correlations of brain and skull dysmorphologies estimated as Procrustes distances at **(A)** PD3, **(B)** PD14, and **(C)** PD29. Brain and skull shape covariation from a Partial Least Squares (PLS) analysis based on brain and skull landmarks at **(D)** PD3 **(E)** PD14 and **(F)** PD29 presented along with the morphings associated with the negative and positive values of both axes of PLS1. Brain landmarks were placed on the 3D renders of brains obtained from *in vivo* μMRI scans and skull landmarks were placed on the surfaces of 3D renders from *in vivo* μCT scans. Sample size: (PD3) WT = 11, TS = 6, WT Treated = 13, TS Treated = 9; (PD14) WT = 11 (12 dysmorphology), TS = 8, WT Treated = 11, TS Treated = 7; (PD29) WT = 10, TS = 4, WT Treated = 7, TS Treated = 7.

Next, we assessed brain and skull patterns of variation and tested whether deviations from the normal co-variation, induced by genotype and treatment in brain and skull shape were correlated over development. To this aim, for those mice for which both μCT and μMR scans were available, we estimated the deviation from WT-like in brain and skull morphology as the Procrustes distance of each mouse to the average shape of the WT untreated group. Then, we performed a correlation between the Procrustes distances based on brain and skull shape. In this analysis, we included mice from the WT untreated group as this correlation assessed intra-group variation and thus served as an indicator of variance of normal development. In the WT treated, TS untreated and TS treated groups, low Procrustes distances indicated WT morphology or slight dysmorphologies, whereas high Procrustes distances indicated strong deviations, that could be related to increased dysmorphologies (Figures 5A–C).

Regarding the normal developmental trajectory, at PD3, WT untreated mice showed high variation in brain shape (large scattering along the *x*-axis) and low variation in skull shape (restricted scattering along the *y*-axis), indicating that in normal conditions early brain development is more variable and probably under a more active process than skull development (Figure 5A). By PD14, the variation ranges in WT untreated mice switched from the *x*-axis to the *y*-axis, suggesting an adjustment in development at the juvenile stage at which craniofacial development was more variable and predominant after brain development was likely almost completed (Figure 5B). By PD29, the ranges of variation were reduced in both axes, suggesting that both brain and skull development were likely almost completed (Figure 5C).

The regression between brain and skull Procrustes distances was almost flat and not significant in WT untreated mice at PD3 (*R* = −0.13, *P*-value = 0.70) and PD29 (*R* = 0.19, *P*-value = 0.59). On the contrary, the regression in TS untreated mice showed a positive although not significant correlation at PD3 (*R* = 0.75, *P*-value = 0.08), PD14 (*R* = 0.24, *P*-value = 0.57), and PD29 (*R* = 0.37, *P*-value = 0.63), suggesting that TS mice with the most severe brain dysmorphologies also tended to show the most severe skull dysmorphologies (Figure 5A). Moreover, at PD14, TS untreated mice presented an abnormally wide range of variation in the brain as compared to WT untreated mice (Figure 5B), suggesting that between PD3 and PD14, TS mice may lag behind in development. At PD29, normal ranges of variation were reestablished in TS untreated mice (Figure 5C).

GTE-EGCG treated TS mice showed a completely different pattern across development, showing non-significant low and negative correlation between brain and skull deviances from WT average at PD3 (*R* = −0.27, *P*-value = 0.49), PD14 (*R* = −0.12, *P*-value = 0.80), and PD29 (R = −0.45, *P*-value = 0.32). Contrary to the rest of groups, TS treated mice with more distinct brain morphologies at PD29 presented less distinct skull morphologies (Figures 5A–C). Overall, the observed changes may suggest that perinatal 100 mg/kg/day GTE-EGCG affected brain more than skull morphology in treated TS mice.

### Early Brain and Skull Integration Are Altered by Genotype and Perinatal GTE-EGCG Treatment

Finally, we further investigated the integrated development of brain and skull over postnatal stages and how the two systems interact by analyzing their covariation patterns, evaluating how morphological changes in one structure were associated with morphological changes in the other structure.

First, we selected the mice for which we had simultaneous brain and skull images and performed a partial least square (PLS) analysis using the skull and brain 3D landmarks registered in the μCT and μMR scans. The magnitude of the integration between the skull and brain and their covariation pattern in WT untreated mice defined normal integrated development, whereas deviations from this pattern indicated genetic- and/or treatment-dependent alterations. The PLS across stages indicated that morphological integration changed over time in WT untreated mice. WT mice presented a significant brain and skull integration, with moderate to high correlation at PD3 (Figure 5D) (*R* = 0.67, *P*-value = 0.025) and PD14 (Figure 5E) (*R* = 0.75, *P*-value = 0.007). However, at the latest stage of development, covariation was reduced, and brain and skull were no longer tightly integrated, being the correlation coefficient low and non-significant in WT untreated mice at PD29 (Figure 5F) (*R* = 0.44, *P*-value = 0.2). The morphings representing the correlated skull and brain shape changes associated with the negative and positive extremes of PLS1 axes indicated that mice presenting with more brachycephalic skulls also presented with wider and shorter brains. This covariation pattern was present at PD3 and maintained until PD29 (Figures 5D–F).

In TS untreated mice, PLS analyses showed that brain and skull were also highly correlated at PD3 (*R* = 0.83, *P*-value = 0.041), but the magnitude of integration was weaker and not significant by PD14 (Figure 5E) (*R* = 0.60, *P*-value = 0.12) and PD29 (Figure 5F) (*R* = 0.64, *P*-value = 0.36). Despite the large genetic imbalance associated with DS, and the brain and skull morphological dysmorphologies induced by these genotype differences in TS mice (Figures 2C, 4C), WT and TS untreated mice presented similar patterns of covariation, as the correlated shape changes were consistent with those in WT mice and the slopes indicating how the brain and skull covary were parallel (Figures 5D–F), even when they gradually separated over development. These results indicated that trisomy did not alter the normal covariation patterns in TS mice (Figure 5F).

GTE-EGCG induced alterations in both the magnitude of the integration and the covariation pattern between the brain and skull. At PD3, the strength of the integration in TS treated mice was lower and not significant (*R* = 0.61, *P*-value = 0.081) as compared to TS untreated mice (*R* = 0.83, *P*-value = 0.041). Indeed, the pattern of covariation was deviated, as the slope of TS treated mice was not parallel to the other groups and mice with more elongated skulls presented with slightly shorter and wider brains instead (Figure 5D). However, this deviation in the brain and skull covariation pattern was reestablished in TS treated mice at PD14 and PD29, as the slopes of TS treated and untreated mice were parallel at these stages (Figures 5E,F). In WT untreated mice, the integration was significant at PD14 (*R* = 0.75, *P*-value = 0.007), however, in WT treated mice the integration was weak and not significant (*R* = 0.32, *P*-value = 0.32) and GTE-EGCG slightly deviated the slope at PD14 and PD29 (Figures 5E,F). Overall, these results showed that GTE-EGCG treatment slightly modulated the morphological integration patterns between the brain and skull.

### Delayed Acquisition of Developmental Traits in Ts65Dn Mice With Mixed Effects of Perinatal GTE-EGCG Treatment

**Figure 6:**
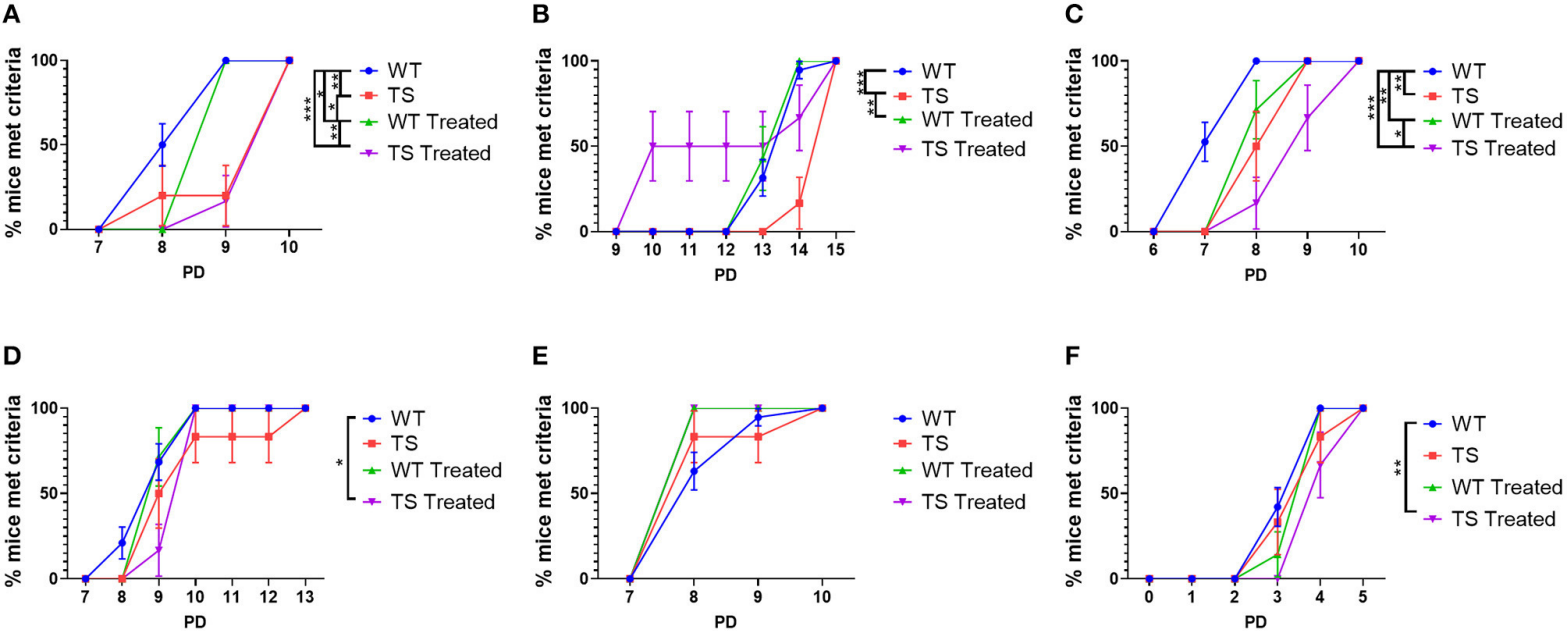
Acquisition of developmental neurobehavioral traits in wild-type and Ts65Dn mice, and effects of GTE-EGCG treatment. **(A)** Incisor eruption, **(B)** eye opening, **(C)** tactile orientation, **(D)** vibrissae placing, **(E)** blast response and **(F)** pinna detachment are represented as a cumulative incidence graph at each postnatal days (PD). Data are presented as percentage of mice that achieved the milestones ± standard error of the mean. Sample size: WT = 19 (16 incisor eruption), TS = 6 (5 incisor eruption), WT Treated = 7, TS Treated = 6. Statistical significance, evaluated using a logrank test, is indicated as ^*^p < 0.05; ^**^p < 0.01; ^***^p < 0.001.

To investigate whether treatment effects were limited to anatomical structures or may also modulate functional developmental traits, we performed a battery of developmental tests from PD1 to PD15 to evaluate the appearance of developmental landmarks and neurobehavioral reflexes in TS and WT mice with and without treatment (Figures 6A–F). TS untreated mice presented a significant delay in day of appearance of the incisors (*P*-value = 0.0028, logrank tests) and opening of the eyes (*P*-value = 0.0007), as well as in the acquisition of tactile orientation reflex (*P*-value = 0.0015) (Figures 6A–C). Treatment with GTE-EGCG accelerated the opening of the eyes that occurred earlier in TS treated mice than in WT untreated mice and the acquisition of the blast response in both WT and TS treated mice, even though these results did not reach significance (Figures 6B,E). However, GTE-EGCG significantly delayed incisor eruption (*P*-value = 0.0235) and tactile orientation (*P*-value = 0.0036) in WT treated mice as compared to WT untreated mice (Figures 6A,C).

## Discussion

Complex genetic disorders such as Down syndrome (DS) affect multiple body systems and produce a constellation of integrated congenital abnormalities, including skeletal and brain alterations leading to delayed neurodevelopment (13, 54). Research of such complex disorders has typically focused on cross-sectional studies at a certain stage of development, commonly using qualitative univariate measurements to investigate each system as an isolated and independent entity, without considering their integrated nature (24, 25, 28–31). This approach has impeded to assess how the genetic and developmental interactions between the affected systems modulate phenotypic outcomes and treatment responses.

### Getting the Full Picture in DS: Integrated Analysis and Treatment of Brain, Skull and Neurobehavioral Development

**Figure 7:**
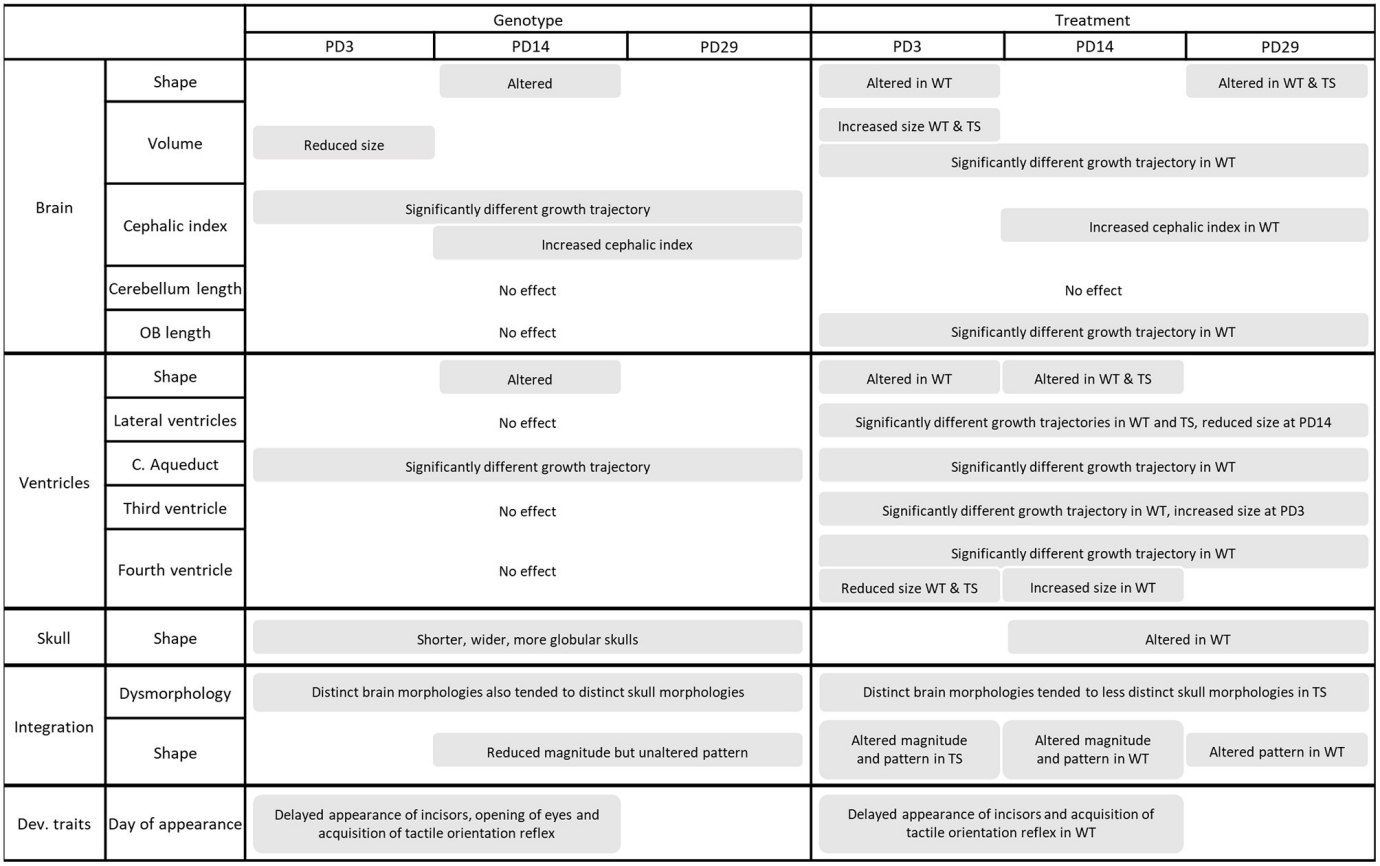
Summary of the main findings of the current article. Each system and parameter analyzed is presented in rows. Genotype and treatment effects are presented in columns at each postnatal day (PD), presented in sub columns. OB length, olfactory bulbs length; C. aqueduct, cerebral aqueduct; Dev. Traits, developmental traits.

We here have developed a novel protocol to characterize the simultaneous development of brain, skeleton, and the acquisition of neurodevelopmental traits in the same mice from birth to juvenile stages. This approach combines (1) a state-of-the-art imaging pipeline using *in vivo* brain MRI and whole-body μCT to visualize the brain and skeletal systems over development, with (2) geometric morphometric methods to quantify shape variation across multiple systems and timepoints; and (3) a battery of developmental tests to evaluate neurobehavioral development. We have optimized imaging protocols and data analysis to statistically compare normal and altered morphological and neurodevelopmental traits of newborn up to juvenile mice, identifying how growth trajectories are altered by genetic disorders and whether these trajectories are reestablished simultaneously in different systems by potential treatments. A summary of the main results can be found in Figure 7.

Our method revealed simultaneous brain and craniofacial abnormalities along with delayed appearance of some neurodevelopmental traits in Ts65Dn (TS) mice. Consistent with previous studies (16, 54, 59, 60) we detected global brain shape alterations in DS, including brachycephaly and reduced brain size. Those alterations were not found at all developmental stages, but interestingly, the most significant dysmorphologies were found at the later stages, supporting the hypothesis that DS is a developmental disorder (61–63). Ventriculomegaly, a trait often associated with DS, has been detected in DS models with the same triplicated genomic region as the Ts65Dn, such as Ts2Cje (15–17, 57, 58), but not in adult Ts65Dn mice (64). Our analyses did not detect ventriculomegaly at early postnatal stages, although a trend toward larger ventricles was observed at PD29. These differences between studies could be explained by (1) technical differences, since we used *in vivo* 3D MR images while previous articles used either ultrasound images, histological tissue sections, *ex vivo* MR or 2D MR images, and (2) differences associated with the developmental stage analyzed, as ours is the first study to evaluate the ventricular volume at early postnatal stages in mice. Moreover, we also evaluated ventricular morphology in a detail never assessed before, detecting an anterior-posterior ventricular bending in TS mice at PD14 and PD29 (Figures 3B,C) consistent with the brachycephaly and craniofacial dysmorphologies reported in our study and typically found in DS (18–20).

Skull malformations were present in TS mice since the earliest stage of postnatal development studied, PD3, whereas brain dysmorphologies generally appeared later, at PD14 and PD29. This is different from the human condition, where brain and craniofacial malformations are simultaneously present prenatally (59) and are maintained at birth (16, 65). Contrary to the most common idea that the brain induces changes in the skull (2, 3), our results indicate that malformations arose first in the skull, and through correlated changes may have indirectly impacted the brain.

The combined use of μMRI and μCT also enabled us to directly investigate the integration pattern of brain and skeletal systems throughout development. Covariation patterns precisely define interdependence among systems and reflect genetic, developmental or functional interaction (11, 12). Our results demonstrated that brain and skull shape covary at PD3 and PD14 in WT untreated mice, indicating that the integration between brain and skull is strong during early stages of postnatal development, when both systems are still growing to achieve their adult size and shape. Our longitudinal analysis showed that the integration of brain and skull lasted until PD29, where the correlation between the two systems faded and became non-significant. This is in line with a previous study showing low brain and skull integration in adult humans (66) and suggests that integration becomes less relevant when the two systems have almost completed their growth.

In trisomic mice, the magnitude of integration was reduced earlier in development, but we did not observe a disrupted brain and skull covariation pattern, indicating that the integration pattern was preserved despite the genetic dosage imbalance associated with DS. This is in line with previous studies in mouse models for Apert and Crouzon syndromes (67), in which despite severe dysmorphologies, the correlation pattern between brain and skull is not altered, thus confirming the high stability of integration in the vertebrate head (7, 9, 12, 68).

Interestingly, perinatal GTE-EGCG supplementation (100 mg/kg/day) modified both the magnitude and the covariation pattern of brain and skull. In WT treated mice, GTE-EGCG altered the integration patterns at PD14 and PD29, and in TS treated mice, it reduced the magnitude of integration between the brain and skull at PD3, a crucial period for head development in which these organs are integrated and grow together to properly accommodate their development. GTE-EGCG treatment no longer showed an effect on the integration patterns at PD14 and PD29, even though the altered correlation between brain and skull dysmorphologies was maintained, suggesting that altering early integration patterns may have downstream effects over development. In summary, our results suggest that the modulatory effects of GTE-EGCG on brain and skull may not be just the direct effect of altering associated signaling pathways in the brain and skeletal cranial tissues, but also to indirect effects induced by alterations in the normal magnitude and pattern of integration, which may explain further secondary dysmorphogenesis in DS. This highlights the important role of early integration of the brain and skull and suggests that altering this integration may lead to further dysregulating effects.

In this study, we assessed the overall effect of GTE-EGCG on development, and confirmed that it can significantly alter brain, skull and neurobehavioral development, as well as the integrated development of the head. Future experiments specifically designed to assess the mechanisms of action should now further elucidate the mechanisms underlying these effects. Reduction of DYRK1A kinase activity is one of the reigning hypotheses behind the mechanism of action of EGCG (24, 69). However, as there are other compounds present in the GTE formulation administered to the mice, more research is required to investigate whether other mechanisms not related to modulation of DYRK1A could be also contributing to the observed effects.

Our results showed that treatment response was variable across treated mice. To ensure that the variation observed in this experiment was representative of biological variation and the natural response to GTE-EGCG treatment, we minimized confounding experimental factors as much as possible. All F1 littermates were bred from the same colony of progenitors, acquired on the same date, and all mice were treated with the same batch of GTE-EGCG. Although it was not possible to ensure that all pups consumed the exact amount of water because we did not administer the treatment via oral gavage to reduce the stress of the pups, we monitored the amount of water consumed by each cage and estimated the average intake per pup, assuming that all pups were drinking similar amounts of water and taking the same dose of treatment. Other factors such as litter effects could also potentially induce some variability in our sample. Since these factors are inevitable in experimental designs and biological samples are inherently variable, our longitudinal approach largely overcomes statistical issues with inter-litter and inter-individual variability as compared to cross-sectional studies, allowing to statistically analyze the growth trajectories of groups of individuals across development.

### Future Perspectives: Further Integrated Research of Developmental Disorders and Modulation

Studies focusing on just one single system and timepoint ignore the effects on other systems at different stages of development. Our results highlight the added value of a longitudinal research approach evaluating the integrated development of multiple systems and their interactions from early to late postnatal stages, as well as the simultaneous modulatory effects of a pharmacological intervention, to reveal the complex etiology of congenital malformations.

In the context of DS, comparing the results from different cross-sectional studies poses a challenge given the high subject-to-subject and across generation variability of the TS65Dn mouse model (34). The advantage of a longitudinal approach is that the same mice are evaluated throughout development, assessing multiple systems simultaneously and increasing the statistical power. Our multimodal approach to investigate the integrated development of brain and skeletal systems in the Ts65Dn model of DS can be further expanded to structural and functional evaluation of other systems, such as lungs and heart, which are also altered in DS and present common signaling pathways (70), but also to other multisystemic disorders such as holoprosencephaly, micro- and macrocephaly, Apert, Pfeiffer and Crouzon craniosynostosis syndromes, investigating each syndrome not only as a compendium of diseases and alterations, but as an integrated system with interrelated development.

## Data Availability Statement

The raw data supporting the conclusions of this article will be made available by the authors, without undue reservation.

## Ethics Statement

The animal study was reviewed and approved by Animal Ethics Committee of KU Leuven.

## Author Contributions

NM-A and GV: conceptualization, supervision, and funding acquisition. WG, NM-A, and GV: methodology. SL, RG, and FM: formal analysis. SL, JA, and JW: investigation. NM-A, GV, MD, UH, and JS: resources. SL, NM-A, and GV: data curation, writing—original draft preparation, and visualization. SL, NM-A, MD, and GV: writing—review and editing. GV: project administration. All authors have read and agreed to the published version of the manuscript.

## Funding

This research was funded by research grants from CRG Awards BrainFace, the Jerome Lejeune Foundation (3M180477), KU Leuven BOF (C24/17/061 and STG/15/024), Grup de Recerca Consolidat en Antropologia Biològica, GREAB (2017 SGR 1630) and a 2-year doctoral fellowship from the Marie-Marguerite Delacroix Foundation to SL.

## Conflict of Interest

The authors declare that the research was conducted in the absence of any commercial or financial relationships that could be construed as a potential conflict of interest.

## Publisher's Note

All claims expressed in this article are solely those of the authors and do not necessarily represent those of their affiliated organizations, or those of the publisher, the editors and the reviewers. Any product that may be evaluated in this article, or claim that may be made by its manufacturer, is not guaranteed or endorsed by the publisher.

## References

[1] Marcucio RS Young NM Hu D Hallgrimsson B. Mechanisms that underlie co-variation of the brain and face. Genesis. (2011) 49:177–89. 10.1002/dvg.20710 21381182 PMC3086711

[2] Richtsmeier JT Flaherty K. Hand in glove: brain and skull in development and dysmorphogenesis. Acta Neuropathol. (2013) 125:469–89. 10.1007/s00401-013-1104-y 23525521 PMC3652528

[3] Gondré-Lewis MC Gboluaje T Reid SN Lin S Wang P Green W. The human brain and face: mechanisms of cranial, neurological and facial development revealed through malformations of holoprosencephaly, cyclopia and aberrations in chromosome 18. J Anat. (2015) 227:255–67. 10.1111/joa.12343 26278930 PMC4560560

[4] Klingenberg CP. Studying morphological integration and modularity at multiple levels: concepts and analysis. Philos Transact R Soc B Biol Sci. (2014) 369:20130249. 10.1098/rstb.2013.0249 25002695 PMC4084535

[5] Mitteroecker P Bookstein F. The conceptual and statistical relationship between modularity and morphological integration. Syst Biol. (2007) 56:818–36. 10.1080/10635150701648029 17934997

[6] Hallgrimsson B Lieberman DE Young NM Parsons T Wat S. Evolution of covariance in the mammalian skull. Tinkering. (2006) 284:164–90. 10.1002/9780470319390.ch12 17710853

[7] Anjali G. Cranial modularity shifts during mammalian evolution. Am Nat. (2006) 168:270–80. 10.1086/505758 16874636

[8] Hallgrímsson B Brown JJY Ford-Hutchinson AF Sheets HD Zelditch ML Jirik FR. The brachymorph mouse and the developmental-genetic basis for canalization and morphological integration. Evol Dev. (2006) 8:61–73. 10.1111/j.1525-142X.2006.05075.x 16409383

[9] Hallgrímsson B Lieberman DE Liu W Ford-Hutchinson AF Jirik FR. Epigenetic interactions and the structure of phenotypic variation in the cranium. Evol Dev. (2007) 9:76–91. 10.1111/j.1525-142X.2006.00139.x 17227368

[10] Richtsmeier J DeLeon V. Morphological integration of the skull in craniofacial anomalies. Orthodont Craniofac Res. (2009) 12:149–58. 10.1111/j.1601-6343.2009.01448.x 19627516 PMC2804975

[11] Klingenberg CP. Evolution and development of shape: integrating quantitative approaches. Nat Rev Genet. (2010) 11:623–35. 10.1038/nrg2829 20697423

[12] Martínez-Abadías N Heuzé Y Wang Y Jabs EW Aldridge K Richtsmeier JT. FGF/FGFR signaling coordinates skull development by modulating magnitude of morphological integration: evidence from Apert Syndrome mouse models. PLoS ONE. (2011) 6:e26425. 10.1371/journal.pone.0026425 22053191 PMC3203899

[13] Wiseman FK Alford KA Tybulewicz VLJ Fisher EMC. Down syndrome–recent progress and future prospects. Human Mol Genet. (2009) 18:R75–83. 10.1093/hmg/ddp010 19297404 PMC2657943

[14] Lott IT Dierssen M. Cognitive deficits and associated neurological complications in individuals with Down's syndrome. Lancet Neurol. (2010) 9:623–33. 10.1016/S1474-4422(10)70112-5 20494326

[15] Pearlson GD Breiter SN Aylward EH Warren AC Grygorcewicz M Frangou S. MRI brain changes in subjects with Down syndrome with and without dementia. Dev Med Child Neurol. (1998) 40:326–34. 10.1111/j.1469-8749.1998.tb15384.x 9630260

[16] Patkee PA Baburamani AA Kyriakopoulou V Davidson A Avini E Dimitrova R. Early alterations in cortical and cerebellar regional brain growth in Down Syndrome: an *in vivo* fetal and neonatal MRI assessment. NeuroImage Clin. (2020) 25:102139. 10.1016/j.nicl.2019.102139 31887718 PMC6938981

[17] Movsas TZ Spitzer AR Gewolb IH. Ventriculomegaly in very-low-birthweight infants with Down syndrome. Dev Med Child Neurol. (2016) 58:1167–71. 10.1111/dmcn.13191 27357997

[18] Richtsmeier JT Baxter LL Reeves RH. Parallels of craniofacial maldevelopment in down syndrome and Ts65Dn mice. Dev Dyn. (2000) 217:137–45. 10.1002/(SICI)1097-0177(200002)217:2<137::AID-DVDY1>3.0.CO 10706138

[19] Suri S Tompson BD Cornfoot L. Cranial base, maxillary and mandibular morphology in Down syndrome. Angle Orthodont. (2010) 80:861–9. 10.2319/111709-650.1 20578856 PMC8939010

[20] Starbuck JM Llambrich S Gonzàlez R Albaigès J Sarlé A Wouters J. Green tea extracts containing epigallocatechin-3-gallate modulate facial development in Down syndrome. Sci Rep. (2021) 11:4715. 10.1038/s41598-021-83757-1 33633179 PMC7907288

[21] Blazek JD Abeysekera I Li J Roper RJ. Rescue of the abnormal skeletal phenotype in Ts65Dn Down syndrome mice using genetic and therapeutic modulation of trisomic Dyrk1a. Hum Mol Genet. (2015) 24:5687–96. 10.1093/hmg/ddv284 26206885

[22] de la Torre R de Sola S Hernandez G Farré M Pujol J Rodriguez J. Safety and efficacy of cognitive training plus epigallocatechin-3-gallate in young adults with Down's syndrome (TESDAD): a double-blind, randomised, placebo-controlled, phase 2 trial. Lancet Neurol. (2016) 15:801–10. 10.1016/S1474-4422(16)30034-5 27302362

[23] Abeysekera I Thomas J Georgiadis TM Berman AG Hammond MA Dria KJ. Differential effects of Epigallocatechin-3-gallate containing supplements on correcting skeletal defects in a Down syndrome mouse model. Mol Nutr Food Res. (2016) 60:717–26. 10.1002/mnfr.201500781 26748562 PMC4828301

[24] De la Torre R De Sola S Pons M Duchon A de Lagran MM Farré M. Epigallocatechin-3-gallate, a DYRK1A inhibitor, rescues cognitive deficits in Down syndrome mouse models and in humans. Mol Nutr Food Res. (2014) 58:278–88. 10.1002/mnfr.201300325 24039182

[25] Guedj F Sébrié C Rivals I Ledru A Paly E Bizot JC. Green tea polyphenols rescue of brain defects induced by overexpression of DYRK1A. PLoS ONE. (2009) 4:e4606. 10.1371/journal.pone.0004606 19242551 PMC2645681

[26] Goodlett CR Stringer M LaCombe J Patel R Wallace JM Roper RJ. Evaluation of the therapeutic potential of Epigallocatechin-3-gallate (EGCG) via oral gavage in young adult Down syndrome mice. Sci Rep. (2020) 10:10426. 10.1038/s41598-020-67133-z 32591597 PMC7319987

[27] Gu Y Moroy G Paul JL Rebillat AS Dierssen M de la Torre R. Molecular rescue of Dyrk1A overexpression alterations in mice with Fontup(®) dietary supplement: role of green tea catechins. Int J Mol Sci. (2020) 1404:1–19. 10.3390/ijms21041404 32092951 PMC7073110

[28] McElyea SD Starbuck JM Tumbleson-Brink DM Harrington E Blazek JD Ghoneima A. Influence of prenatal EGCG treatment and Dyrk1a dosage reduction on craniofacial features associated with Down syndrome. Hum Mol Genet. (2016) 25:4856–69. 10.1093/hmg/ddw309 28172997 PMC6049609

[29] Stringer M Abeysekera I Dria KJ Roper RJ Goodlett CR. Low dose EGCG treatment beginning in adolescence does not improve cognitive impairment in a Down syndrome mouse model. Pharmacol Biochem Behav. (2015) 138:70–9. 10.1016/j.pbb.2015.09.002 26363314

[30] Souchet B Duchon A Gu Y Dairou J Chevalier C Daubigney F. Prenatal treatment with EGCG enriched green tea extract rescues GAD67 related developmental and cognitive defects in Down syndrome mouse models. Sci Rep. (2019) 9:3914. 10.1038/s41598-019-40328-9 30850713 PMC6408590

[31] Stagni F Giacomini A Emili M Guidi S Ciani E Bartesaghi R. Epigallocatechin gallate: a useful therapy for cognitive disability in Down syndrome? Neurogenesis. (2017) 4:e1270383. 10.1080/23262133.2016.1270383 28203607 PMC5293319

[32] Chu KO Wang CC Chu CY Choy KW Pang CP Rogers MS. Uptake and distribution of catechins in fetal organs following *in utero* exposure in rats. Hum Reprod. (2006) 22:280–7. 10.1093/humrep/del353 16959805

[33] Chu KO Wang CC Chu CY Chan KP Rogers MS Choy KW. Pharmacokinetic studies of green tea catechins in maternal plasma and fetuses in rats. J Pharm Sci. (2006) 95:1372–81. 10.1002/jps.20594 16625654

[34] Shaw PR Klein JA Aziz NM Haydar TF. Longitudinal neuroanatomical and behavioral analyses show phenotypic drift and variability in the Ts65Dn mouse model of Down syndrome. Dis Models Mech. (2020) 13:1–16. 10.1242/dmm.046243 32817053 PMC7522024

[35] Motulsky HJ Brown RE. Detecting outliers when fitting data with nonlinear regression - a new method based on robust nonlinear regression and the false discovery rate. BMC Bioinform. (2006) 7:123. 10.1186/1471-2105-7-123 16526949 PMC1472692

[36] Dryden IL Mardia KV. Statistical Shape Analysis. Chichester: Wiley (1998).

[37] James Rohlf F Marcus LF. A revolution morphometrics. Trends Ecol Evol. (1993) 8:129–32. 10.1016/0169-5347(93)90024-J 21236128

[38] Klingenberg CP. MorphoJ: an integrated software package for geometric morphometrics. Mol Ecol Resour. (2011) 11:353–7. 10.1111/j.1755-0998.2010.02924.x 21429143

[39] Hallgrimsson B Percival CJ Green R Young NM Mio W Marcucio R. Morphometrics, 3D imaging, and craniofacial development. Curr Top Dev Biol. (2015) 115:561–97. 10.1016/bs.ctdb.2015.09.003 26589938 PMC5299999

[40] R Core Team. R: A Language and Environment for Statistical Computing. 3.6.2 ed. Vienna: R Foundation for Statistical Computing (2019).

[41] Kassambara A. ggpubr: 'ggplot2' Based Publication Ready Plots: R Package Version 0.4.0. (2020). Available online at: https://CRAN.R-project.org/package=ggpubr

[42] Rohlf FJ Corti M. Use of two-block partial least-squares to study covariation in shape. Syst Biol. (2000) 49:740–53. 10.1080/106351500750049806 12116437

[43] Klingenberg CP. Morphometric integration and modularity in configurations of landmarks: tools for evaluating a priori hypotheses. Evol Dev. (2009) 11:405–21. 10.1111/j.1525-142X.2009.00347.x 19601974 PMC2776930

[44] Dierssen M Fotaki V Martínez de Lagrán M Gratacós M Arbonés M Fillat C. Neurobehavioral development of two mouse lines commonly used in transgenic studies. Pharmacol Biochem Behav. (2002) 73:19–25. 10.1016/S0091-3057(02)00792-X 12076721

[45] Reeves RH Irving NG Moran TH Wohn A Kitt C Sisodia SS. A mouse model for Down syndrome exhibits learning and behaviour deficits. Nat Genet. (1995) 11:177–84. 10.1038/ng1095-177 7550346

[46] Davisson MT Schmidt C Akeson EC. Segmental trisomy of murine chromosome 16: a new model system for studying Down syndrome. Progr Clin Biol Res. (1990) 360:263–80. 2147289

[47] Herault Y Delabar JM Fisher EMC Tybulewicz VLJ Yu E Brault V. Rodent models in Down syndrome research: impact and future opportunities. Dis Model Mech. (2017) 10:1165–86. 10.1242/dmm.029728 28993310 PMC5665454

[48] Duchon A Raveau M Chevalier C Nalesso V Sharp AJ Herault Y. Identification of the translocation breakpoints in the Ts65Dn and Ts1Cje mouse lines: relevance for modeling Down syndrome. Mamm Genome. (2011) 22:674–84. 10.1007/s00335-011-9356-0 21953411 PMC3224224

[49] Reinholdt LG Ding Y Gilbert GJ Czechanski A Solzak JP Roper RJ. Molecular characterization of the translocation breakpoints in the Down syndrome mouse model Ts65Dn. Mamm Genome. (2011) 22:685–91. 10.1007/s00335-011-9357-z 21953412 PMC3505986

[50] MuñizMoreno MdM Brault V Birling M-C Pavlovic G Herault Y. Modeling down syndrome in animals from the early stage to the 4.0 models and next. In: Dierssen M, editor. Progress in Brain Research. Amsterdam: Elsevier (2020). p. 91–143. 10.1016/bs.pbr.2019.08.001 32057313

[51] Aziz NM Guedj F Pennings JLA Olmos-Serrano JL Siegel A Haydar TF. Lifespan analysis of brain development, gene expression and behavioral phenotypes in the Ts1Cje, Ts65Dn and Dp(16)1/Yey mouse models of Down syndrome. Dis Models Mech. (2018) 11:dmm031013. 10.1242/dmm.031013 29716957 PMC6031353

[52] Blazek JD Gaddy A Meyer R Roper RJ Li J. Disruption of bone development and homeostasis by trisomy in Ts65Dn Down syndrome mice. Bone. (2011) 48:275–80. 10.1016/j.bone.2010.09.028 20870049 PMC3021595

[53] Gupta M Dhanasekaran AR Gardiner KJ. Mouse models of Down syndrome: gene content and consequences. Mammalian Genome. (2016) 27:538–55. 10.1007/s00335-016-9661-8 27538963 PMC5471624

[54] Rodrigues M Nunes J Figueiredo S Martins de Campos A Geraldo AF. Neuroimaging assessment in Down syndrome: a pictorial review. Insights Imaging. (2019) 10:52. 10.1186/s13244-019-0729-3 31111268 PMC6527671

[55] Shapiro BL. Developmental instability of the cerebellum and its relevance to Down syndrome. In: Lubec G, editor. Protein Expression in Down Syndrome Brain. Vienna: Springer Vienna (2001). p. 11–34. 10.1007/978-3-7091-6262-0_2 11771737

[56] Baxter LL Moran TH Richtsmeier JT Troncoso J Reeves RH. Discovery and genetic localization of Down syndrome cerebellar phenotypes using the Ts65Dn mouse. Hum Mol Genet. (2000) 9:195–202. 10.1093/hmg/9.2.195 10607830

[57] Raveau M Nakahari T Asada S Ishihara K Amano K Shimohata A. Brain ventriculomegaly in Down syndrome mice is caused by Pcp4 dose-dependent cilia dysfunction. Hum Mol Genet. (2017) 26:923–31. 10.1093/hmg/ddx007 28069794

[58] Ishihara K Amano K Takaki E Shimohata A Sago HJ Epstein C. Enlarged brain ventricles and impaired neurogenesis in the Ts1Cje and Ts2Cje mouse models of Down Syndrome. Cerebr Cortex. (2009) 20:1131–43. 10.1093/cercor/bhp176 19710359

[59] Guihard-Costa A-M Khung S Delbecque K Ménez F Delezoide A-L. Biometry of face and brain in fetuses with trisomy 21. Pediatr Res. (2006) 59:33–8. 10.1203/01.pdr.0000190580.88391.9a 16326987

[60] Aldridge K Reeves RH Olson LE Richtsmeier JT. Differential effects of trisomy on brain shape and volume in related aneuploid mouse models. Am J Med Genet Part A. (2007) 143A:1060–70. 10.1002/ajmg.a.31721 17431903 PMC3246902

[61] Holtzman DM Santucci D Kilbridge J Chua-Couzens J Fontana DJ Daniels SE. Developmental abnormalities and age-related neurodegeneration in a mouse model of Down syndrome. Proc Natl Acad Sci USA. (1996) 93:13333–8. 10.1073/pnas.93.23.13333 8917591 PMC24093

[62] Lockrow JP Fortress AM Granholm A-CE. Age-related neurodegeneration and memory loss in Down Syndrome. Curr Gerontol Geriatr Res. (2012) 2012:463909. 10.1155/2012/463909 22545043 PMC3318235

[63] Perluigi M Di Domenico F Buttterfield DA. Unraveling the complexity of neurodegeneration in brains of subjects with Down syndrome: insights from proteomics. Proteom Clin Appl. (2014) 8:73–85. 10.1002/prca.201300066 24259517 PMC3965623

[64] Duchon A del Mar Muniz Moreno M Martin Lorenzo S Silva de Souza MP Chevalier C Nalesso V. Multi-influential genetic interactions alter behaviour and cognition through six main biological cascades in Down syndrome mouse models. Hum Mol Genet. (2021) 30:771–88. 10.1093/hmg/ddab012 33693642 PMC8161522

[65] Fischer-Brandies H Schmid RG Fischer-Brandies E. Craniofacial development in patients with Down's syndrome from birth to 14 years of age. Eur J Orthodont. (1986) 8:35–42. 10.1093/ejo/8.1.35 2937647

[66] Bruner E Amano H de la Cuétara JM Ogihara N. The brain and the braincase: a spatial analysis on the midsagittal profile in adult humans. J Anat. (2015) 227:268–76. 10.1111/joa.12355 26200138 PMC4560561

[67] Motch Perrine SM Stecko T Neuberger T Jabs EW Ryan TM Richtsmeier JT. Integration of brain and skull in prenatal mouse models of Apert and Crouzon Syndromes. Front Hum Neurosci. (2017) 11:369. 10.3389/fnhum.2017.00369 28790902 PMC5525342

[68] Porto A de Oliveira FB Shirai LT De Conto V Marroig G. The evolution of modularity in the mammalian skull I: morphological integration patterns and magnitudes. Evol Biol. (2009) 36:118–35. 10.1007/s11692-008-9038-332220100

[69] Duchon A Herault Y. DYRK1A, a dosage-sensitive gene involved in neurodevelopmental disorders, is a target for drug development in Down Syndrome. Front Behav Neurosci. (2016) 10:104. 10.3389/fnbeh.2016.00104 27375444 PMC4891327

[70] Arron JR Winslow MM Polleri A Chang C-P Wu H Gao X. NFAT dysregulation by increased dosage of DSCR1 and DYRK1A on chromosome 21. Nature. (2006) 441:595–600. 10.1038/nature04678 16554754

