# Supplementary material for: Multimodal in vivo Imaging of the Integrated Postnatal Development of Brain and Skull and Its Co-modulation With Neurodevelopment in a Down Syndrome Mouse Model
Source: Front Med (Lausanne). 2022 Feb 11;9:815739. doi: 10.3389/fmed.2022.815739 (PMC8874331; doi:10.3389/fmed.2022.815739)
Supplement: Supplementary file 1 [file Data_Sheet_1.docx]

Supplementary Material

# Supplementary Figure


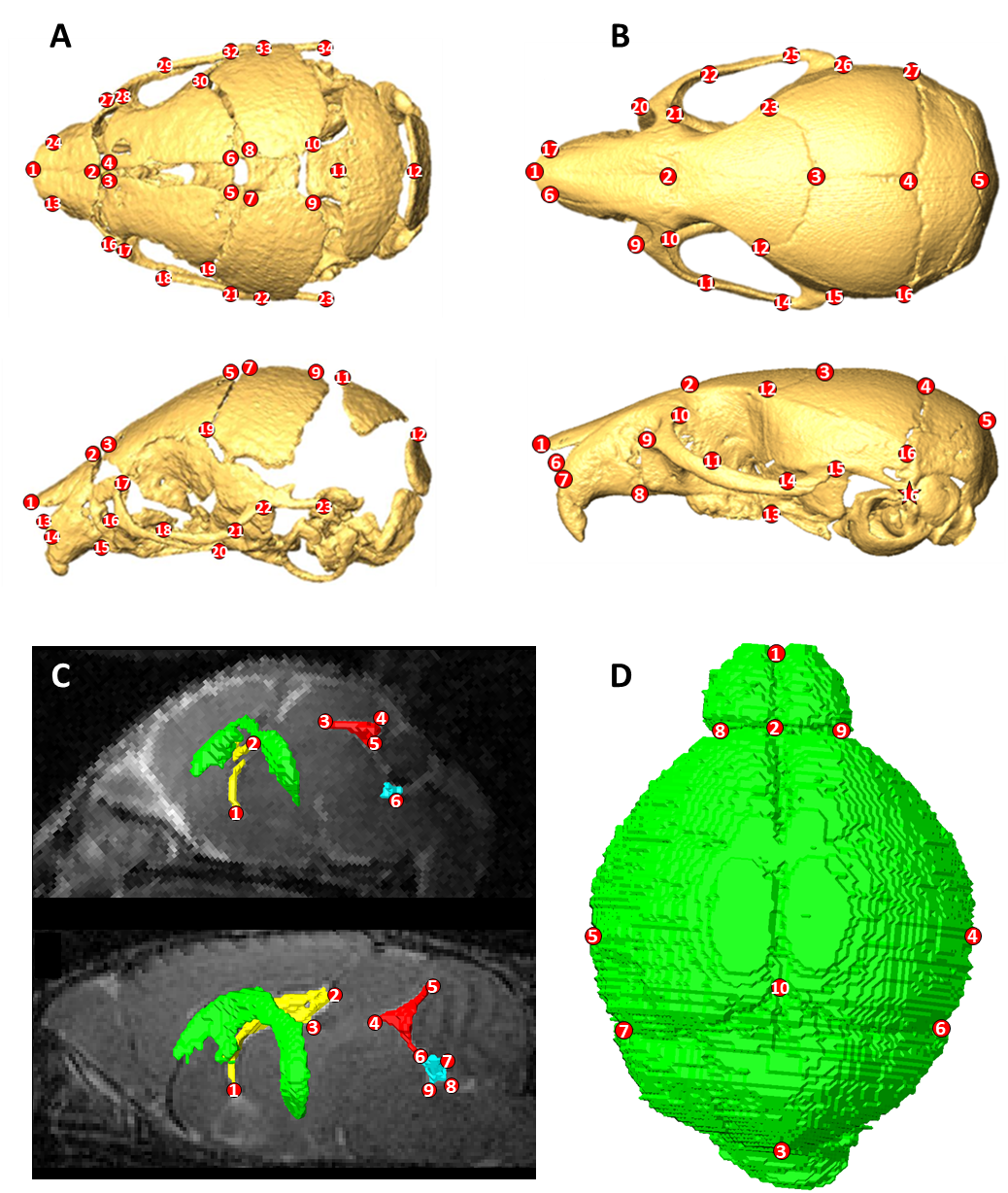


**Supplementary Figure S1. Set of anatomical landmarks used to characterize the shape of the skull, brain and ventricles. (A)** Set of 34 landmarks characterizing skull shape at PD3 from a 3D reconstruction of a µCT scan. **(B)** Set of 27 landmarks characterizing skull shape at PD14 and PD29 from a 3D reconstruction of a µCT scan. **(C)** Set of 6 landmarks used to characterize ventricle shape from the sagittal plane of a µMRI scan at PD3 (top) and 9 landmarks used at PD14 and 29 (bottom). **(D)** Set of 10 landmarks characterizing brain shape from a 3D reconstruction of a µMRI scan. See Supplementary Tables S3 to S5 for precise anatomical definitions.

# Supplementary Tables

|  | PD3 | | | | | PD14 | | | | | PD29 | | | | |
| --- | --- | --- | --- | --- | --- | --- | --- | --- | --- | --- | --- | --- | --- | --- | --- |
|  | WT | TS | WT Treated | TS Treated | WT | | TS | WT Treated | TS Treated | WT | | TS | WT Treated | TS Treated |  |
| Brain volume | 12 | 8 | 13 | 9 | 17 | | 9 | 13 | 8 | 14 | | 4 | 14 | 7 |  |
| Cephalic index | 12 | 8 | 13 | 9 | 17 | | 9 | 13 | 8 | 14 | | 4 | 14 | 7 |  |
| Cerebellum length | 12 | 8 | 12 | 9 | 17 | | 9 | 13 | 8 | 14 | | 4 | 14 | 6 |  |
| Olfactory bulbs length | 10 | 8 | 11 | 8 | 17 | | 9 | 13 | 8 | 14 | | 4 | 14 | 7 |  |
| Brain shape | 12 | 8 | 13 | 9 | 17 | | 9 | 13 | 8 | 14 | | 4 | 14 | 7 |  |
| Lateral ventricles volume | 11 | 8 | 11 | 7 | 8 | | 4 (1 outlier) | 12 (1 outlier) | 8 | 14 | | 4 | 13 (1 outlier) | 7 |  |
| Cerebral aqueduct volume | 7 (3 outliers) | 7 (1 outlier) | 6 (5 outliers) | 6 | 10 | | 7 | 13 | 8 | 13 (1 outlier) | | 4 | 14 | 7 |  |
| Third ventricle volume | 10 | 6 | 12 | 7 | 5 | | 5 | 13 | 8 | 11 | | 3 | 14 | 7 |  |
| Fourth ventricle volume | 11 | 8 | 12 | 7 | 9 | | 7 | 13 | 8 | 11 | | 4 | 14 | 7 |  |
| Ventricles shape | 10 | 8 | 12 | 6 | 10 | | 6 | 15 | 8 | 14 | | 4 | 14 | 7 |  |
| Skull shape | 12 | 6 | 15 | 9 | 17 | | 8 | 13 | 7 | 14 | | 7 | 7 | 8 |  |
| Skull and brain integration | 11 | 6 | 13 | 9 | N/A | | N/A | N/A | N/A | 10 | | 4 | 7 | 7 |  |
| Skull and brain dysmorphology | 11 | 6 | 13 | 9 | N/A | | N/A | N/A | N/A | 10 | | 4 | 7 | 7 |  |
| Incisor eruption | 16* | 5* | 7* | 6* | N/A | | N/A | N/A | N/A | N/A | | N/A | N/A | N/A |  |
| Eye opening | 19* | 6* | 7* | 6* | N/A | | N/A | N/A | N/A | N/A | | N/A | N/A | N/A |  |
| Tactile orientation | 19* | 6* | 7* | 6* | N/A | | N/A | N/A | N/A | N/A | | N/A | N/A | N/A |  |
| Vibrissae placing | 19* | 6* | 7* | 6* | N/A | | N/A | N/A | N/A | N/A | | N/A | N/A | N/A |  |
| Blast response | 19* | 6* | 7* | 6* | N/A | | N/A | N/A | N/A | N/A | | N/A | N/A | N/A |  |
| Pinna detachment | 19* | 6* | 7* | 6* | N/A | | N/A | N/A | N/A | N/A | | N/A | N/A | N/A |  |

**Supplementary Table S1. Sample size for each experiment and developmental stage.** Number of outliers per test (identified by the ROUT method) are indicated in brackets. Note that tests marked with an asterisk were performed daily from postnatal day 1 to postnatal day 15, and the sample sizes thus represent the entire duration of the testing. N/A indicates that the data is not available.

| Stage | Source energy (Kv) | Filter | Current (µA) | Exposure time (ms) | Averages | Step increment (˚) | Total angle (˚) | Time | Voxel size (µm^3^) | Radiation dose |
| --- | --- | --- | --- | --- | --- | --- | --- | --- | --- | --- |
| PD3 | 35 | Al 0.5mm | 500 | 180 | 3 | 1 | 180º | 3 min | 51.7 | 25 mGy |
| PD14 | 35 | Al 0.5mm | 500 | 80 | 3 | 1 | 180º | 3 min | 51.7 | 110 mGy |
| PD29 | 55 | Al 1mm | 700 | 80 | 2 | 1 | 360º | 3 min | 51.7 | 249 mGy |

**Supplementary Table S2. µCT scanning parameters used at each stage.**

| Landmark number | Anatomical definition |
| --- | --- |
| 1 | Anterior-most point on the intersection of the olfactory bulbs |
| 2 | Intersection point between the midline of the cerebrum and the olfactory bulbs |
| 3 | Posterior-most point on the cerebellum |
| 4 | Lateral-most point on the cerebrum (right) |
| 5 | Lateral-most point on the cerebrum (left) |
| 6 | Lateral-most point on the intersection between the cerebellum and the cerebrum (right) |
| 7 | Lateral-most point on the intersection between the cerebellum and the cerebrum (left) |
| 8 | Lateral-most point on the intersection between the cerebrum and the olfactory bulbs (left) |
| 9 | Lateral-most point on the intersection between the cerebrum and the olfactory bulbs (right) |
| 10 | Intersection point between the midline of the cerebrum and the cerebellum |
| 11 | Posterior-most point of the medullary hindbrain (only visible at PD3) |

**Supplementary Table S3. Anatomical definitions of brain landmarks.** The ratio of the Euclidean distance between landmarks number 4 and 5 and landmarks number 1 and 3 (1 and 11 at PD3) was used to calculate the cephalic index. The length of the olfactory bulbs and the length of the cerebellum were determined as the Euclidean distance between landmarks number 1 and 2 and 10 and 3 respectively.

| Landmark number at PD3 | Landmark number at PD14 and PD29 | Anatomical definition |
| --- | --- | --- |
| 1 | 1 | Anterior-most point on the third ventricle |
| N/A | 2 | Most posterior-dorsal point on the third ventricle |
| N/A | 3 | Most posterior-ventral point on the third ventricle |
| 3 | 4 | Anterior-most point on the cerebral aqueduct |
| 4 | 5 | Most posterior-dorsal point on the cerebral aqueduct |
| N/A | 6 | Intersection point between the cerebral aqueduct and the fourth ventricle |
| N/A | 7 | Most posterior-dorsal point on the fourth ventricle |
| N/A | 8 | Posterior-most point on the fourth ventricle |
| 6 | 9 | Most posterior-ventral point on the fourth ventricle |
| 2 | N/A | Posterior-most point on third ventricle |
| 5 | N/A | Most posterior-ventral point on cerebral aqueduct |

**Supplementary Table S4. Anatomical definition of brain ventricles landmarks.**

| Landmark number at PD3 | Landmark number at PD14 and PD29 | Anatomical definition |
| --- | --- | --- |
| 1 | 1 | Tip of the nasal bone |
| N/A | 2 | Intersection of nasal and frontal bones |
| N/A | 3 | Intersection of frontal and parietal bones |
| N/A | 4 | Intersection of parietal and interparietal bones |
| N/A | 5 | Intersection of interparietal and occipital bones |
| 13 | 6 | Anterior-most point on intersection of premaxillae and nasal bones (left) |
| 14 | 7 | Center of alveolar ridge over maxillary incisor (left) |
| 15 | 8 | Most inferior point on premaxilla-maxilla suture (left) |
| 16 | 9 | Anterior notch on frontal process lateral to infraorbital fissure (left) |
| 17 | 10 | Intersection of frontal process of maxilla with frontal and lacrimal bones (left) |
| 18 | 11 | Intersection of zygomatic process of maxilla with zygoma (left) |
| 19 | 12 | Frontal-squamosal intersection at temporal crest (left) |
| 20 | 13 | Intersection of maxilla and sphenoid on inferior alveolar (left) |
| 21 | 14 | Intersection of zygoma with zygomatic process of temporal (left) |
| N/A | 15 | Intersection of squamosal body to zygomatic process of squamosal (left) |
| N/A | 16 | Intersection of parietal, temporal and occipital bones (left) |
| 24 | 17 | Anterior-most point on intersection of premaxillae and nasal bones (right) |
| 25 | 18 | Center of alveolar ridge over maxillary incisor (right) |
| 26 | 19 | Most inferior point on premaxilla-maxilla suture (right) |
| 27 | 20 | Anterior notch on frontal process lateral to infraorbital fissure (right) |
| 28 | 21 | Intersection of frontal process of maxilla with frontal and lacrimal bones (right) |
| 29 | 22 | Intersection of zygomatic process of maxilla with zygoma (right) |
| 30 | 23 | Frontal-squamosal intersection at temporal crest (right) |
| 31 | 24 | Intersection of maxilla and sphenoid on inferior alveolar (right) |
| 32 | 25 | Intersection of zygoma with zygomatic process of temporal (right) |
| N/A | 26 | Intersection of squamosal body to zygomatic process of squamosal (right) |
| N/A | 27 | Intersection of parietal, temporal and occipital bones (right) |
| 2 | N/A | Posterior-most point on the nasal bone at the midline |
| 3 | N/A | Anterior-most point on the frontal bone at the midline (left) |
| 4 | N/A | Anterior-most point on the frontal bone at the midline (right) |
| 5 | N/A | Posterior-most point on the frontal bone at the midline (left) |
| 6 | N/A | Posterior-most point on the frontal bone at the midline (right) |
| 7 | N/A | Anterior-most point on the parietal bone at the midline (left) |
| 8 | N/A | Anterior-most point on the parietal bone at the midline (right) |
| 9 | N/A | Maximum curvature point on the posterior part of the parietal bone (left) |
| 10 | N/A | Maximum curvature point on the posterior part of the parietal bone (right) |
| 11 | N/A | Anterior-most point on the interparietal bone at the midline |
| 12 | N/A | Anterior-most point on the occipital bone at the midline |
| 22 | N/A | Joining of squamosal body to zygomatic process of squamosal (left) |
| 23 | N/A | Tip of the post-tympanic hook (left) |
| 33 | N/A | Joining of squamosal body to zygomatic process of squamosal (right) |
| 34 | N/A | Tip of the post-tympanic hook (right) |

**Supplementary Table S5. Anatomical definition of skull landmarks.**

| PD3 | TS_Treated | TS_Untreated | WT_Treated |
| --- | --- | --- | --- |
| TS_Untreated | 0.5764 |  |  |
| WT_Treated | 0.2836 | 0.1184 |  |
| WT_Untreated | 0.0861 | 0.5553 | **0.0260** |
| PD14 | TS_Treated | TS_Untreated | WT_Treated |
| TS_Untreated | 0.4710 |  |  |
| WT_Treated | **0.0212** | **0.0016** |  |
| WT_Untreated | **0.0133** | **0.0095** | 0.1005 |
| PD29 | TS_Treated | TS_Untreated | WT_Treated |
| TS_Untreated | **0.0112** |  |  |
| WT_Treated | 0.2739 | **<.0001** |  |
| WT_Untreated | **0.0163** | 0.0519 | **0.0093** |

**Supplementary Table S6. *P*-values resulting from the permutation tests (10,000 permutation rounds) for the Procrustes distances among groups based on brain shape:**

| PD3 | TS_Treated | TS_Untreated | WT_Treated |
| --- | --- | --- | --- |
| TS_Untreated | 0.2985 |  |  |
| WT_Treated | 0.9747 | 0.1017 |  |
| WT_Untreated | 0.0570 | 0.1148 | **0.0166** |
| PD14 | TS_Treated | TS_Untreated | WT_Treated |
| TS_Untreated | **0.0384** |  |  |
| WT_Treated | **0.0265** | **0.0130** |  |
| WT_Untreated | **<.0001** | **0.0001** | **0.0202** |
| PD29 | TS_Treated | TS_Untreated | WT_Treated |
| TS_Untreated | 0.3866 |  |  |
| WT_Treated | **0.0435** | 0.1853 |  |
| WT_Untreated | **0.0258** | 0.5796 | 0.2677 |

**Supplementary Table S7. *P*-values resulting from the permutation tests (10,000 permutation rounds) for the Procrustes distances among groups for the ventricular shape:**

| PD3 | TS_Treated | TS_Untreated | WT_Treated |
| --- | --- | --- | --- |
| TS_Untreated | 0.3120 |  |  |
| WT_Treated | **0.0003** | **0.0437** |  |
| WT_Untreated | **<.0001** | **0.0042** | 0.0519 |
| PD14 | TS_Treated | TS_Untreated | WT_Treated |
| TS_Untreated | 0.2963 |  |  |
| WT_Treated | **0.0019** | **0.0003** |  |
| WT_Untreated | **<.0001** | **<.0001** | **<.0001** |
| PD29 | TS_Treated | TS_Untreated | WT_Treated |
| TS_Untreated | 0.1501 |  |  |
| WT_Treated | **0.0026** | **0.0027** |  |
| WT_Untreated | **<.0001** | **0.0002** | **0.0281** |

**Supplementary Table S8. *P*-values resulting from the permutation tests (10,000 permutation rounds) for the Procrustes distances among groups for the skull shape:**
